# MicroRNA-126 regulates Hypoxia-Inducible Factor-1α which inhibited migration, proliferation, and angiogenesis in replicative endothelial senescence

**DOI:** 10.1038/s41598-019-43689-3

**Published:** 2019-05-14

**Authors:** Matilde Alique, Guillermo Bodega, Chiara Giannarelli, Julia Carracedo, Rafael Ramírez

**Affiliations:** 1 Departamento Biología de Sistemas, Facultad de Medicina y Ciencias de la Salud, Universidad de Alcalá (IRYCIS), Alcalá de Henares, Madrid Spain; 2 Departamento de Biomedicina y Biotecnología, Facultad de Biología, Química y Ciencias Ambientales, Universidad de Alcalá. Alcalá de Henares, Madrid, Spain; 3 Cardiovascular Research Center, One Gustave L. Levy Place, New York, NY USA; 4 Institute for Genomics and Multiscale Biology, One Gustave L. Levy Place, New York, NY USA; 5 Precision Immunology Institute, Icahn School of Medicine at Mount Sinai, One Gustave L. Levy Place, New York, NY USA; 6 Departamento de Genética, Fisiología y Microbiología, Facultad de Biología, Universidad Complutense de Madrid, Madrid, Spain; 7 Instituto de Investigación Sanitaria Hospital 12 de Octubre (imas12), Madrid, Spain

**Keywords:** Cell growth, Cell growth, Cell division, Cell division, Ageing

## Abstract

Whereas a healthy endothelium maintains physiological vascular functions, endothelial damage contributes to the development of cardiovascular diseases. Endothelial senescence is the main determinant of endothelial dysfunction and thus of age-related cardiovascular disease. The objective of this study is to test the involvement of microRNA-126 and HIF-1α in a model of replicative endothelial senescence and the interrelationship between both molecules in this *in vitro* model. We demonstrated that senescent endothelial cells experience impaired tube formation and delayed wound healing. Senescent endothelial cells failed to express HIF-1α, and the microvesicles released by these cells failed to carry HIF-1α. Of note, HIF-1α protein levels were restored in HIF-1α stabilizer-treated senescent endothelial cells. Finally, we show that microRNA-126 was downregulated in senescent endothelial cells and microvesicles. With regard to the interplay between microRNA-126 and HIF-1α, transfection with a microRNA-126 inhibitor downregulated HIF-1α expression in early passage endothelial cells. Moreover, while HIF-1α inhibition reduced tube formation and wound healing closure, microRNA-126 levels remained unchanged. These data indicate that HIF-1α is a target of miRNA-126 in protective and reparative functions, and suggest that their therapeutic modulation could benefit age-related vascular disease.

## Introduction

The vascular endothelium, the epithelial layer that lines the inner surface of blood and lymphatic vessels, forms a nearly 1-kg organ and consists of approximately 1–6 × 10^13^ cells. Endothelial cells have important physiological functions, and their dysfunction can contribute to several pathological conditions, including cardiovascular disease. Endothelial dysfunction contributes to the development of nearly all types of vascular diseases, such as hypertension, coronary artery disease, peripheral vascular disease, chronic heart failure, diabetes, and chronic kidney failure^1–3^.

The vascular endothelium has specialized functions. Angiogenesis consists of the sprouting of new capillaries from existing vessels to form functional vascular networks in wound healing and response to tissue ischemia (e.g., peripheral artery disease and chronic angina) but also contributes to pathological processes (e.g., cancer, atherosclerosis) when this response is inappropriate^4,5^. Endothelial cell migration is known to be an essential step in angiogenesis^6^. Physiological angiogenesis is highly regulated during development and wound repair^7^, and its dysregulation is associated with various pathological disorders, including age-related macular degeneration, rheumatoid arthritis, tumor progression, and metastasis^8^. The regulation of blood vessel formation is fundamental to many physiological and pathological processes, and angiogenesis is a major area with regard to developing novel therapeutic approaches for diseases, from ischemia to cancer^9^.

Aging is the primary unmodifiable cardiovascular risk factor. Oxidative stress^10^, disruption of cell-cell junctions^11^, dysfunction of endothelial progenitor cells, vascular inflammation or activation of a specific genetic program are all processes involved in vascular aging^12^. Endothelial senescence (ES) has been associated with the initiation or progression of cardiovascular diseases (CVD). ES is linked to vascular aging^13^ or age-related CVD^14^ such as vascular calcification^15^, yet the cellular and molecular mechanisms involved are not fully understood^16^. Several experimental models, such as the Hayflick replicative senescence model^17^, have been useful in identifying the cellular and molecular changes that occur during cellular senescence and aging.

Hypoxia-inducible factor 1 (HIF-1) is an oxygen-dependent heterodimeric (comprising α and β subunits) transcriptional activator that is primarily responsible for adapting cells to hypoxic stress^18^. The target genes of HIF-1 regulate angiogenesis, cell proliferation and survival, and glucose and iron metabolism. HIF-1α concentrations in healthy, well-oxygenated tissue and cells are tightly regulated by constant protein degradation^19^. On the other hand, protein chaperone heat shock protein 90 (Hsp90)^20^ binding stabilizes HIF-1α, preventing its degradation^21^. HIF-1 is suspected to mediate ES, yet no mechanistic evidence supporting these observations exists.

Microvesicles (MVs) are a heterogeneous population of endogenous cell-derived membrane vesicles that are released by many cell types (eg, blood circulating and endothelial cells), with a particle diameter of 100–1000 nm and are shed into the circulation under physiological (homeostasis)^22^ and also in pathological conditions, but to a greater extent in the latter^23^. MVs are critical in intercellular communication due to their capacity to transfer of their biological content (proteins, lipids, and nucleic acids) between cells^24^. MVs mediate physiological and pathological processes, including coagulation, reticulocyte maturation, and angiogenesis^22–24^. MVs circulating levels are elevated in pathological conditions characterized by endothelial dysfunction^2,25^.

MicroRNAs (miRNAs) are non-coding endogenous RNAs (approx. 22 nt) that regulate physiological endothelial cell functions (angiogenesis and wound repair) as well as vascular inflammation in response to pathophysiologic stimuli^26^ by targeting specific mRNAs^27^. MicroRNA-126 (miR-126) is one of the principal regulators of developmental angiogenesis^26^, endothelial proliferation, migration and network vessel formation *in vitro*^28^. Recent findings suggest that miR-126 secreted by endothelial cells is transferred to vascular smooth muscle cells^29^, possibly carried by MVs released by these cells. However, no studies have examined the mechanistic interactions between miR-126 and HIF-1α in the regulation of endothelial cell function or their involvement in ES or thus in the initiation and progression of CVD, prompting us to identify the dynamic interplay between HIF-1α and miR-126, and the role of miR-126 in determining HIF-1α levels.

To test the hypothesis that HIF-1α and miR-126 are involved in ES, we analyzed their expression in endothelial cells using a well-validated and robust model of replicative senescence^17^. Then, we examined the possible mechanistic relationship between miR-126 and HIF-1α and measured HIF-1 and miRNA-126 content in MVs that were released from early passage and senescent endothelial cells.

## Results

### Primary HUVECs with over 96 population doublings display characteristics of senescence

**Figure 1:**
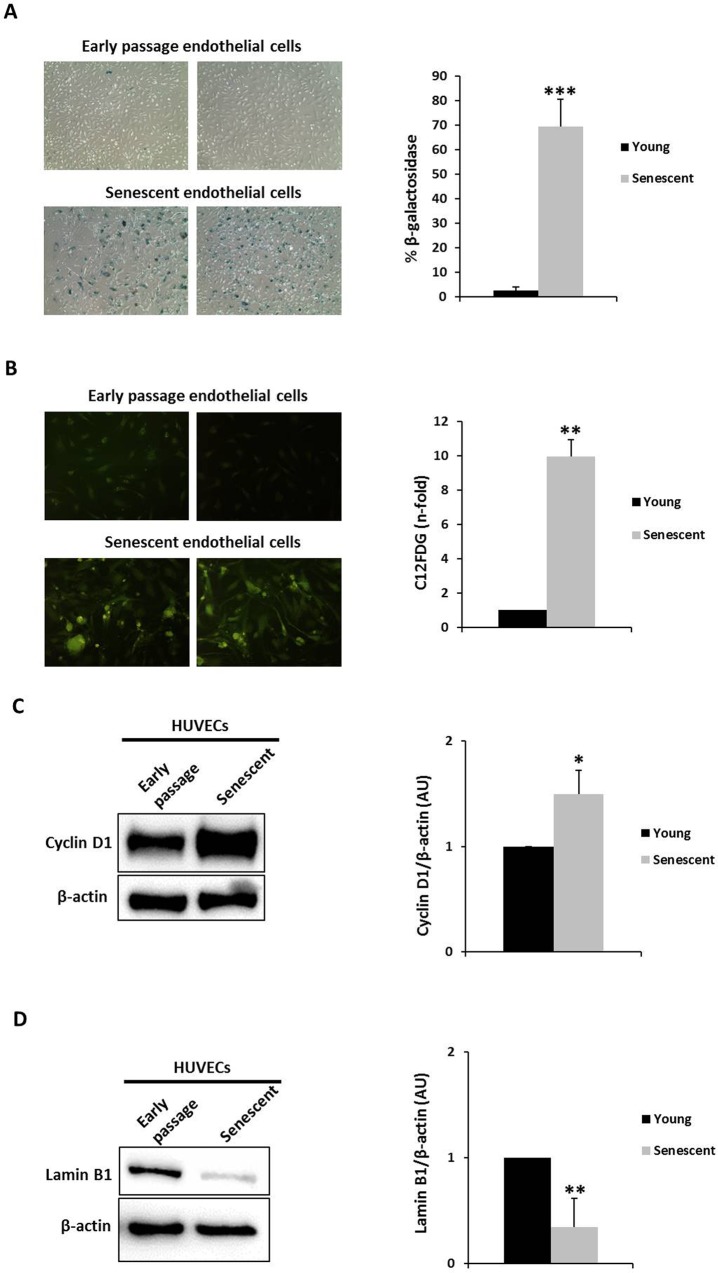
HUVECs senescence markers. HUVECs develop a senescence phenotype with increasing passage number *in vitro*. The percentage of senescent HUVECs at different passages was determined by senescence-associated β-galactosidase staining (**A**) and C12FDG fluorescence staining (**B**). The data represent means ± SD and are expressed as a percentage of total cells and fold induction respectively with respect to control values (early passage cells). Early passage endothelial cells, n = 6; senescent endothelial cells (n = 6); 10 random fields/each; magnification, x100. (**C**) Cyclin D1 and (**D**) Lamin B1 representative Western blots in early passage and senescent HUVECs pools. Equal protein loading was confirmed probing with β-actin. The graphs present densitometric band analysis normalized to β-actin in arbitrary units (AU). The data represent means ± SD and are expressed as fold induction with respect to control values (early passage cells). Early passage endothelial cells n = 3 pools; senescent endothelial cells. n = 3 pools. *p < 0.05, **p < 0.01 and ***p < 0.001. Early passage *vs*. senescent HUVECs cells. In the figure graphs, the early passage is called young.

At population doubling (PD) level of 20, HUVECs (between passages 2 and 8) showed less than 3% (2.67 ± 1.46%) senescence-associated, beta-galactosidase (SA-β-gal)-stained cells (termed early passage endothelial cells in Fig. 1A). Sister cultures of these replicating endothelial cells continued to undergo serial passaging until replicative senescence was reached at PD > 96 (between passages 27 and 38). These replicative senescent endothelial cells assumed the typical flattened and enlarged morphology, with over 69% (69.36 ± 11.30%) being SA-β-gal-positive in the monolayer (termed senescent endothelial cells in Fig. 1A).

Senescence was also measured using the fluorogenic substrate C12FDG, which is cleaved by β-galactosidase, generating a fluorescent product that is well retained by the cells. Early passage and senescent endothelial cells were stained with C12FDG (Fig. 1B) to confirm the results using the traditional SA-β-gal staining protocol (using X-gal as the substrate for β-gal). As expected, senescent endothelial cells showed greater C12FDG fluorescence.

To determine the senescent status of endothelial cells, Cyclin D1 and Lamin B1 proteins were measured by Western blot. Senescent endothelial cells showed Cyclin D1 upregulation (Fig. 1C) compared with early passage endothelial cells, whereas Lamin B1 (Fig. 1D) levels were lower. Thus, these new cellular markers of senescence were modulated in our replicative senescence model in HUVECs. To further validate our model, other typical markers of senescence (early markers of DNA damage-induced senescence), such as p53 and p16, were tested^30,31^. Senescent HUVECs increased p53 and p16 expression by Western blot (Supplemental Fig. 1). These data confirm that new and conventional cellular markers of senescence fluctuate in our HUVEC model.

### Senescent HUVECs have altered endothelial function

**Figure 2:**
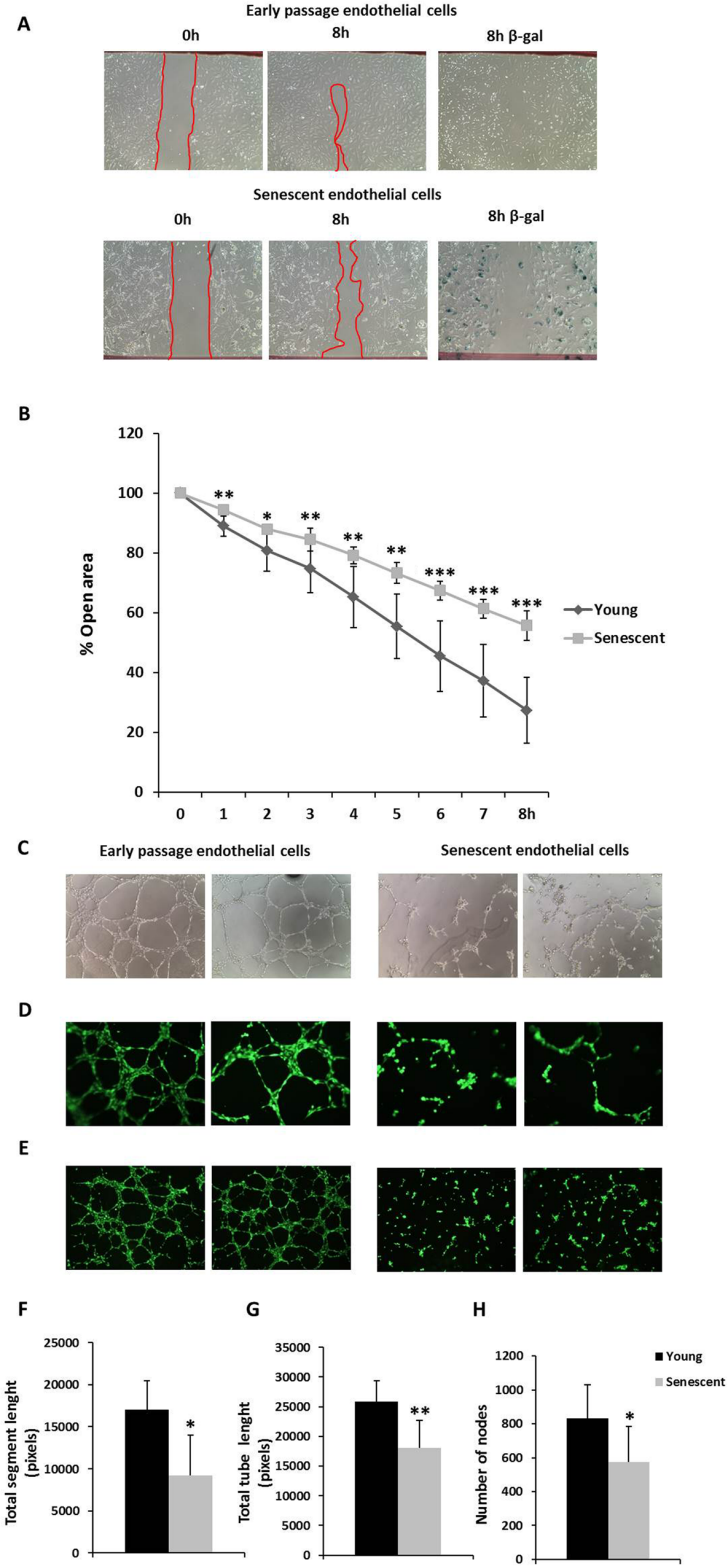
Wound healing in HUVECs monolayers. (**A**) Representative photomicrographs of early passage and senescent HUVECs monolayers 8 hours after wounding. β-galactosidase staining is showed at the final time. Note the flattened morphology and positive senescence-associated SA-β-gal staining of the senescent cells. Red lines indicate the edge of the wound repopulating cells. Magnification 100x. (**B**) Time course of changes in the size of the remaining wound. The data points represent the % open area means ± SD. Early passage endothelial cells, n = 9 in duplicate; senescent endothelial cells, n = 6 in duplicate. *p < 0.05, **p < 0.01 and ***p < 0.001. Early passage *vs*. senescent HUVECs cells at the same time. Endothelial tube formation in HUVECs. The spontaneous formation of capillary-like structures by HUVEC on Matrigel was used to assess angiogenic potential. (**C**) Light microscope pictures and (**D**,**E**) fluorescent microscopy (for HUVECs treated with calcein AM) photomicrographs of early passage and senescent HUVECs seeded on Matrigel-coated wells after 6 h. Early passage HUVECs migrated to form connected tubular networks; senescent HUVECs significantly attenuated network formation. (**F**–**H**) Total segment length, total tube length and the number of nodes were quantitated from photographs of early passage and senescent HUVECs after 6 hours. (**C** and **D**: Magnification: 100x; **E**: Magnification: 40x). Data are expressed as means ± SD. Early passage endothelial cells, n = 10 in triplicate; senescent endothelial cells, n = 6 in quadruple. *p < 0.05, **p < 0.01, Early passage *vs*. senescent HUVECs. In the figure graphs, the early passage is called young.

Endothelial damage is associated with impaired wound healing. To measure its effects on the endothelial aging model *in vitro*, HUVECs migration and proliferation were analyzed by the scratch assay. Inhibition of wound healing was observed in senescent HUVECs monolayers compared with early passage HUVECs cell cultures over 8 hours (Fig. 2). Senescent endothelial cell migration was suppressed, and such cells had a flattened morphology and were positive for SA-β-gal staining at 8 hours (Fig. 2A). Moreover, healing time was measured at different time points over 8 hours and senescent endothelial cells were associated with delayed healing (Fig. 2B). These results show that the migration of senescent endothelial cells, as measured by wound healing, are inhibited versus early passage endothelial cells.

HUVECs tube formation was measured by angiogenesis assay to determine whether senescent endothelial cells maintained their function, in which senescent endothelial cells significantly decreased angiogenesis (Fig. 2). Early passage endothelial cells enhanced tube formation compared with senescent endothelial cells at 6 hours (Fig. 2C–E). We also measured the total segment length, total tube length, and the number of nodes—which all declined significantly in senescent cells versus early passage HUVECs (Fig. 2F–H). Thus, these changes observed during angiogenesis in senescent endothelial cells appear to be associated with aging.

### HIF-1α and Hsp90 expression is lower in senescent endothelial cells and microvesicles

**Figure 3:**
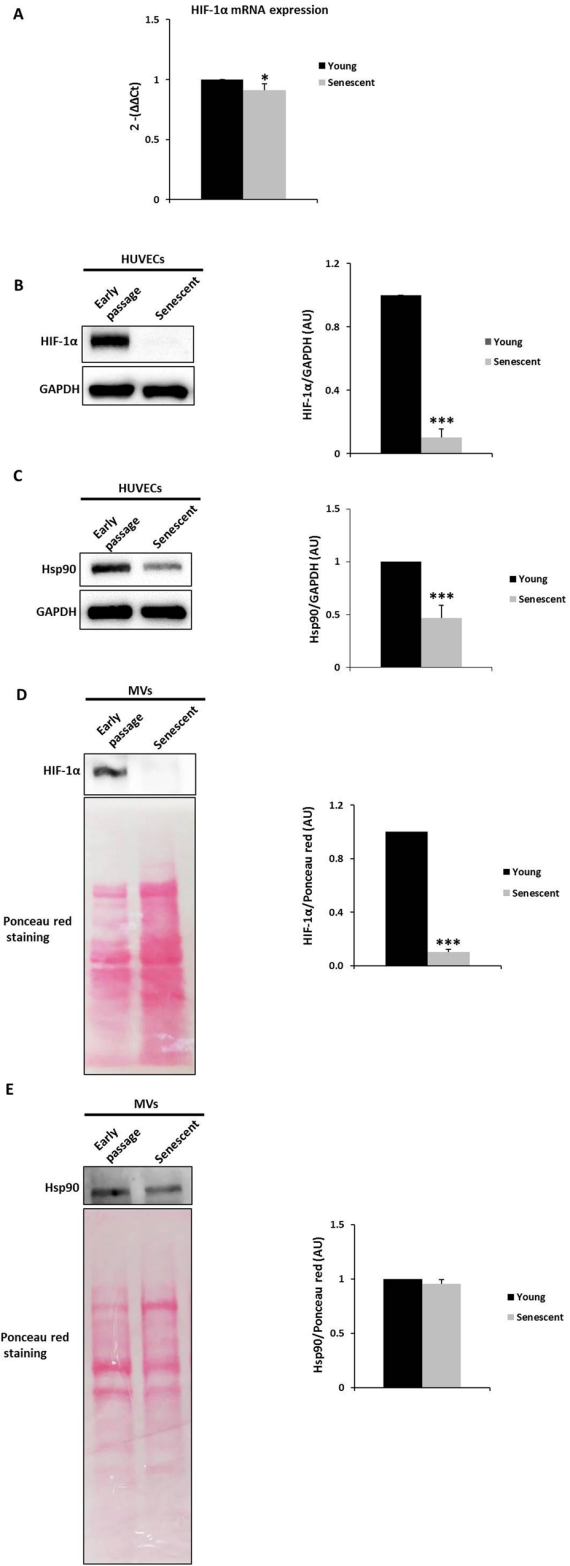
HIF-1α mRNA, and HIF-1α and Hsp90 protein levels in HUVECs. (**A**) qPCR analysis of HIF-1α mRNA levels in early passage and senescent HUVECs pools using the ΔCt method; HPRT1 mRNA was used for normalization. Early passage endothelial cells, n = 3 pools; senescent endothelial cells, n = 3 pools. *p < 0.05. (**B**,**C**) Representative HIF-1α and Hsp90 western blot of early passage and senescent HUVECs pools. Equal protein loading was confirmed probing with GAPDH. The graphs present densitometric band analysis normalized to GAPDH in arbitrary units (AU). Early passage endothelial cells, n = 3 pools; senescent endothelial cells, n = 3 pools. Early passage *vs*. senescent HUVECs. HIF-1α and Hsp90 protein levels of MVs released by HUVECs. (**D**) Representative HIF-1α and (**E**) Hsp90 western blot of early passage and senescent MVs pools. Equal protein loading was confirmed probing with Ponceau red staining. The graphs present densitometric band analysis normalized to Ponceau red staining in arbitrary units (AU). Early passage endothelial MVs, n = 3 pools; senescent endothelial MVs, n = 3 pools. The data represent means ± SD. ***p < 0.001. Early passage *vs*. senescent. In the figure graphs, the early passage is called young.

HIF-1α mRNA and protein levels were examined in both early passage and senescent HUVECs to determine the mechanism of endothelial dysfunction. As shown in Fig. 3A, HIF-1α mRNA was significantly downregulated in senescent versus early passage endothelial cells. Further, HIF-1α protein content was significantly lower in senescent HUVECs (Fig. 3B).

Based on the relationship between heat shock protein 90 (Hsp90) and HIF-1α, Hsp90 was evaluated in early passage and senescent endothelial cells by Western blot to test the possibility that the modulation of HIF-1α in senescent endothelial cells is associated with Hsp90 changes. Paralleling the disappearance of HIF-1α protein in senescent endothelial cells, Hsp90 protein declined significantly in senescent HUVECs (Fig. 3C).

Next, we analyzed HIF-1α levels in MVs that were released from endothelial cells. The results showed that early passage HUVECs-secreted MVs contained significantly more HIF-1α than their senescent counterparts (Fig. 3D). Notably, there were no changes in Hsp90 protein between early passage and senescent MVs (Fig. 3E).

These data clearly demonstrate that replicative endothelial senescence correlates with the loss of HIF-1α in endothelial cells and the MVs that they discharge. Furthermore, Hsp90 expression was significantly downregulated in senescent endothelial cells, implicating it in the disappearance of HIF-1α. On the other hand, there was no change in Hsp90 expression in MVs suggesting that Hsp90 may play a role in the mechanism for MVs loading^32^.

### Deferoxamine mesylate recovers HIF-1α levels in senescent endothelial cells

**Figure 4:**
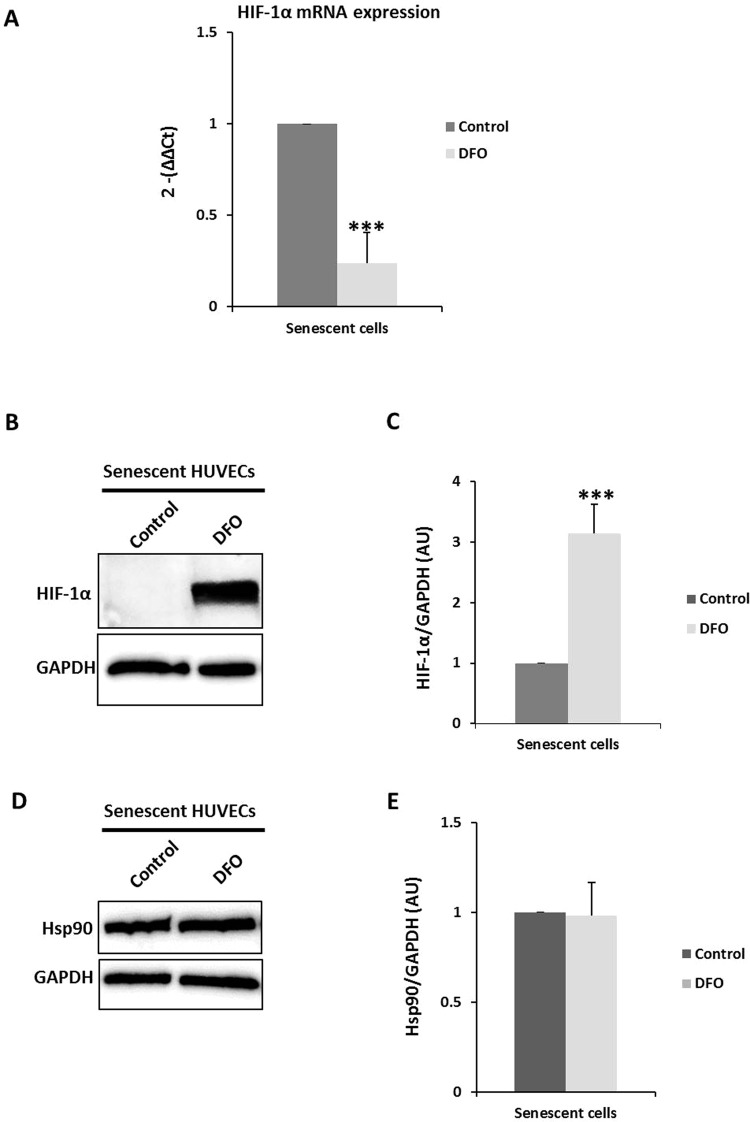
DFO effect on HIF-1α protein in senescent HUVECs. (**A**) qPCR analysis of HIF-1α mRNA in control and DFO-treated senescent HUVECs using the ΔCt method; HPRT1 mRNA was used for normalization. (**B**) Representative HIF-1α and (**D**) Hsp90 western blots in control and DFO-treated (100 µM, 8 hours) senescent HUVECs. Equal protein loading was confirmed probing with GAPDH. (**C**,**E**) The graphs present densitometric band analysis normalized to GAPDH in arbitrary units (AU). The data represent means ± SD. Control *vs*. DFO-treated senescent HUVECs cells. ***p < 0.001 n = 4.

To determine whether the synthesis or degradation of HIF-1α protein in senescent HUVECs is altered, senescent endothelial cells were treated with deferoxamine mesylate (DFO), an agent that stabilizes HIF-1α (Fig. 4). HIF-1α mRNA fell significantly in senescent endothelial cells that were treated with DFO (Fig. 4A), but the HIF-1α losing was reversed by DFO treatment (Fig. 4B,C). This finding suggests that HIF-1α is degraded rapidly in control senescent endothelial cells, an effect that is blunted by the stabilizing effect of DFO and consequent accumulation of HIF-1α in senescent endothelial cells, whereas HIF-1α is degraded rapidly in control senescent endothelial cells. Notably, the accumulation of HIF-1α in DFO-treated senescent HUVECs lowered HIF-1α mRNA levels, perhaps due to a resulting negative feedback mechanism at the transcriptional level through high HIF-1α protein levels. DFO treatment did not alter the Hsp90 content in senescent HUVECs (Fig. 4D,E). These data suggest that higher HIF-1α protein degradation causes the disappearance of HIF-1α protein in senescent endothelial cells independently of Hsp90 protein levels.

**Figure 5:**
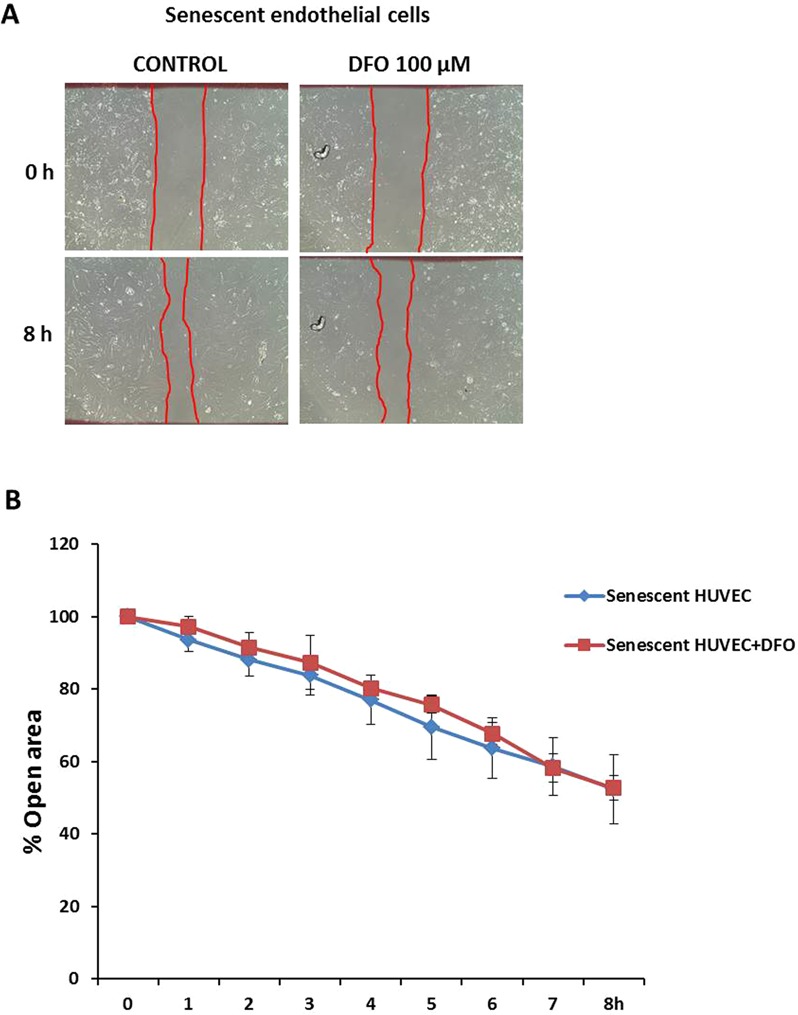
DFO effect on wound healing in senescent HUVECs. (**A**) Representative photomicrographs of senescent and DFO-treated senescent HUVECs cells 8 hours after wounding. Red lines indicate the edge of the wound repopulating cells. Magnification 100x. (**B**) Time course of changes in the size of the remaining wound. The data points represent the % open area means ± SD. n = 4 in duplicate.

We also examined endothelial function in DFO-administered senescent HUVECs by wound-healing assay after up to 8 hours of treatment. As a result, the migration rate of DFO-treated senescent HUVECs was unchanged compared with control senescent HUVECs, suggesting an HIF-1α independent effect (Fig. 5).

### microRNA-126 is decreased in senescent endothelial cells

**Figure 6:**
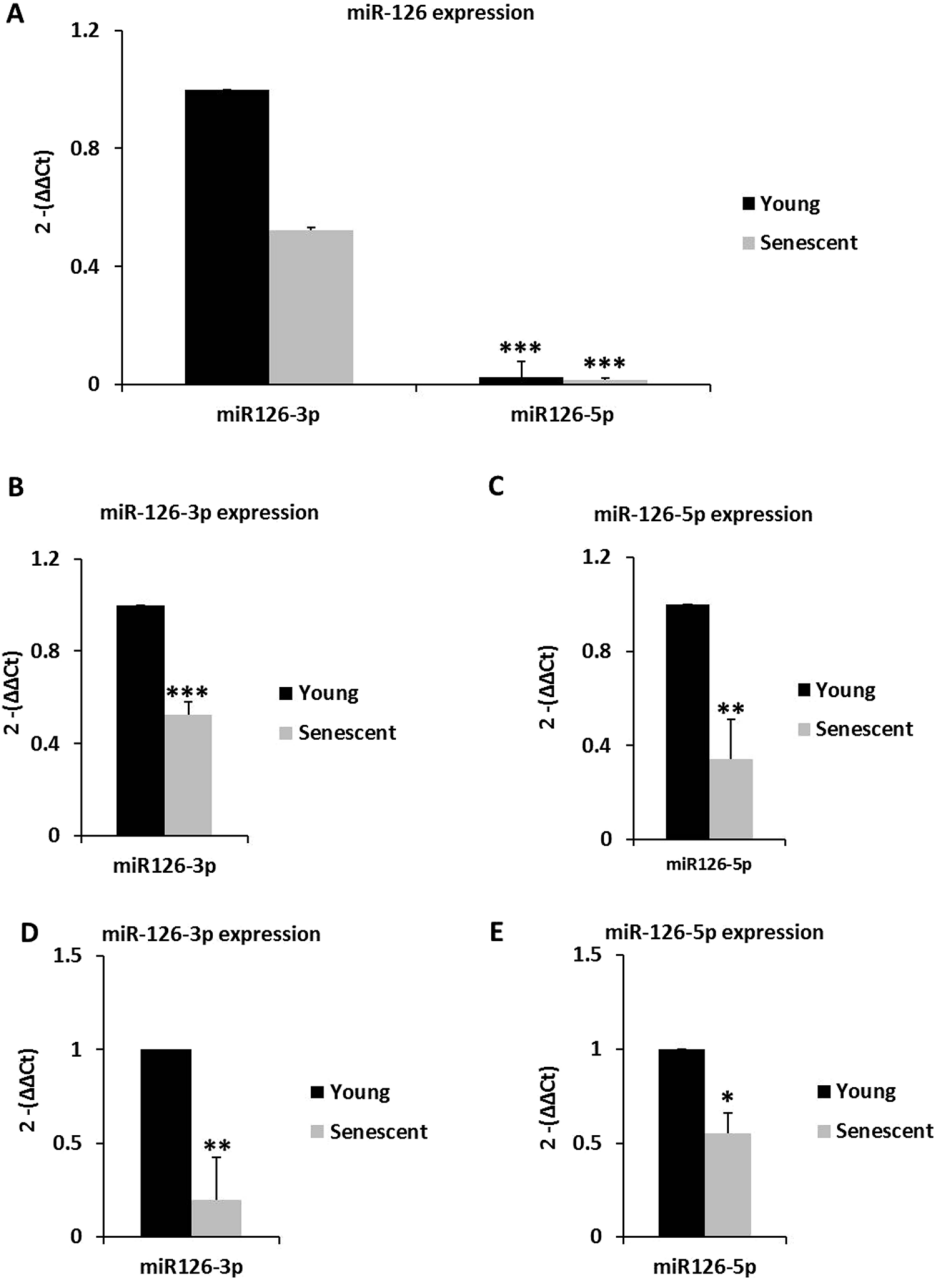
MiR-126 in early passage and senescent HUVECs and MVs. QPCR analysis of miR-126-3p and miR-126-5p was performed in early passage and senescent HUVECs pools (**A**,**B**,**C**) and MVs (**D**,**E**) using the ΔCt method; U6 snRNA was used for normalization in HUVECs. MVs were normalized to a spike in (miR39-3p) levels. (**A**) miR-126-5p expression was lower than miR-126-3p expression in early passage and senescent HUVECs using early passage HUVECs miR-126-3p levels as a control. ***p < 0.001, miR-126-5p *vs* miR-126-3p in early passage HUVECs. (**B**) miR-126-3p and (**C**) miR-126-5p expression was diminished in senescent HUVECs versus early passage HUVECs. (**D**) miR-126-3p and (**E**) miR-126-5p expression were diminished in senescent MVs compared with early passage MVs. Early passage endothelial HUVECs and MVs, n = 3 pools; senescent endothelial HUVECs and MVs, n = 3 pools. The data represent means ± SD. *p < 0.05, **p < 0.01 and ***p < 0.001. Early passage *vs*. senescent HUVECs or MVs. In the figure graphs, the early passage is called young.

MiR-126 is essential for endothelial cell signaling and promotes migration, proliferation, and angiogenesis. miR-126 was measured by qPCR in early passage and senescent HUVECs and MVs. Basal miR-126-5p strand expression was significantly lower compared with miR-126-3p strand in both, early passage and senescent endothelial cells (Fig. 6A). These data are concordant with previous observations that miR-126-5p is less abundant than miR-126-3p in the resting endothelium^26^. The individual analysis of these transcripts showed lower levels of miR-126-3p and miR-126-5p in senescent vs. early passage endothelial cells (Fig. 6B,C). Similarly, senescent HUVECs-secreted MVs contained less miR-126-3p and miR-126-5p (Fig. 6D,E). These data imply that replicative endothelial senescence is associated with a decrease in miR-126-3p and miR-126-5p in senescent HUVECs and MVs from such cultures.

### Effect of HIF-1α inhibition on early passage endothelial cells

**Figure 7:**
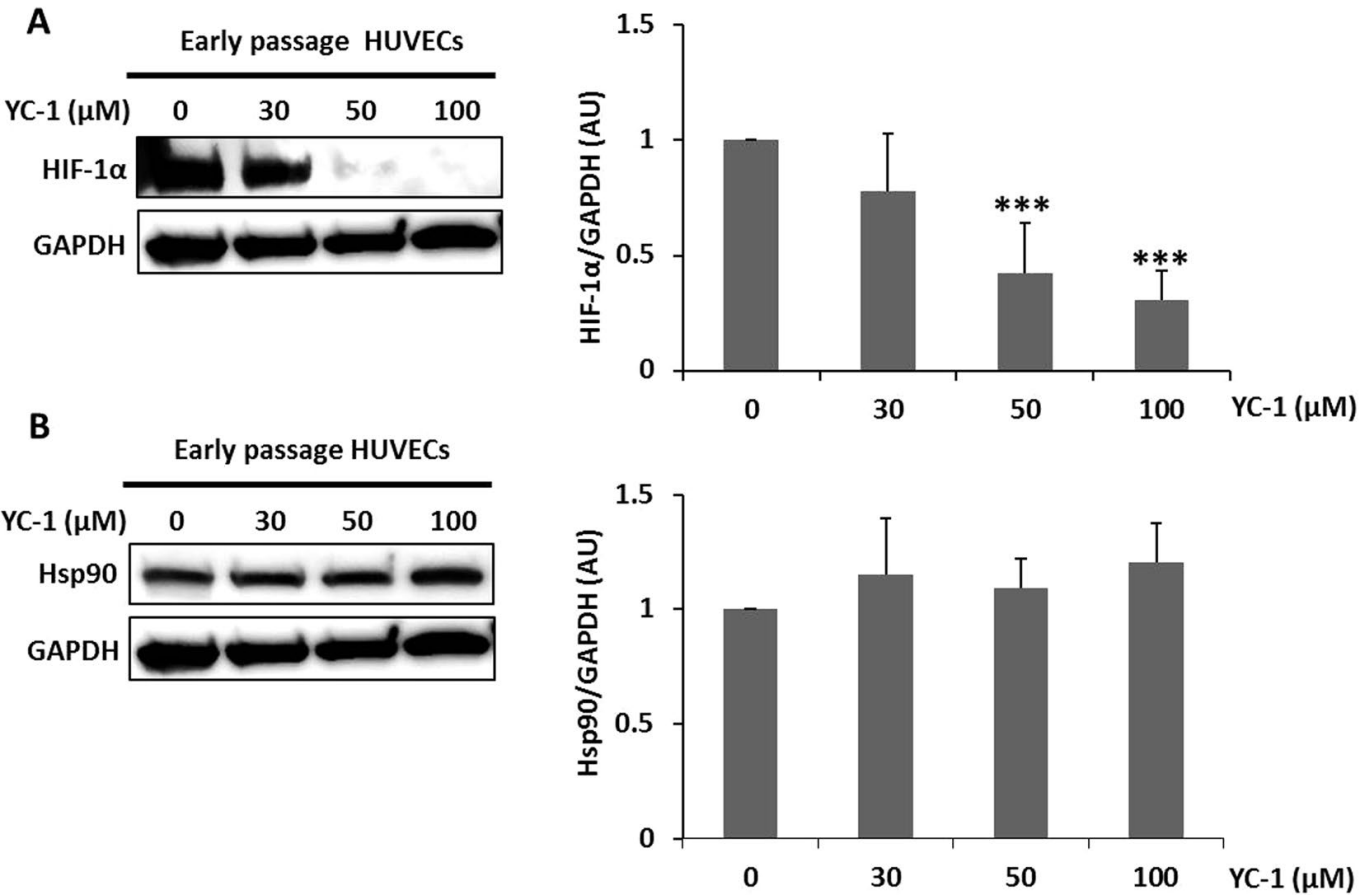
Effect of YC-1 on HIF-1α and Hsp90 proteins in early passage HUVECs. Representative (**A**) HIF-1α and (**B**) Hsp90 protein western blots in early passage HUVECs treated with different doses of YC-1. Equal protein loading was confirmed probing with GAPDH. The graphs present densitometric band analysis normalized to GAPDH in arbitrary units (AU). The data represent means ± SD. n = 4. YC-1 treated vs. Control ***p < 0.001.

We performed functional assays in early passage HUVECs to determine the function of HIF-1α in endothelial damage. Early passage endothelial cells were treated with increasing doses of YC-1 (Abcam), a pharmacological inhibitor of HIF-1α, and examined by Western blot. YC-1 treatment for 16 hours dose-dependently downregulated HIF-1α protein, which was slight at 30 µM YC-1 (Fig. 7A) in early passage HUVECs. At 100 µM, YC-1 was slightly toxic and induced cell death^33^ (cytotoxicity was assessed using Trypan blue in early passage HUVECs; data not shown). YC-1 had no effect on Hsp90 (Fig. 7B).

**Figure 8:**
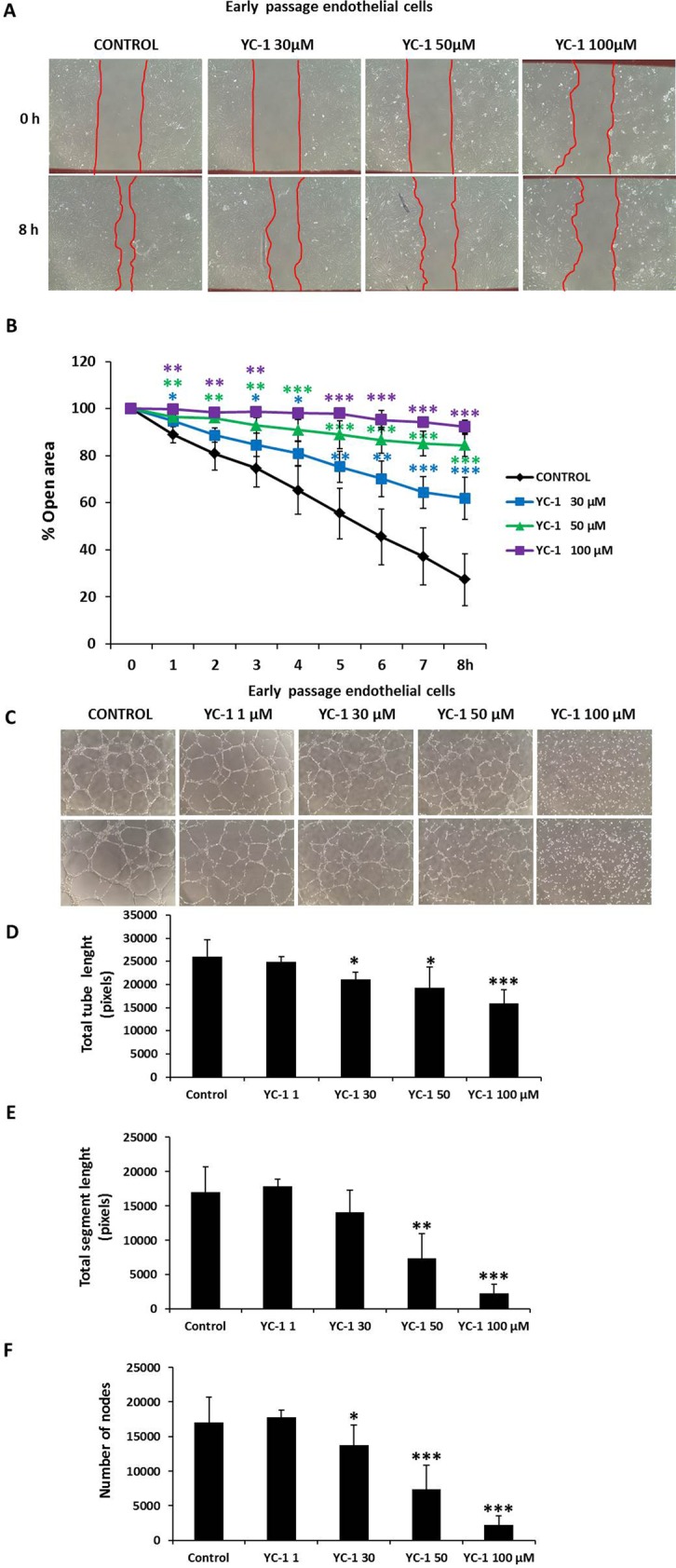
Effect of YC-1 in a scratch assay. (**A**) Representative photomicrographs of cell monolayers 8 hours after wounding. Red lines indicate the edge of the wound repopulating cells. Magnification 100x. (**B**) Time course of changes in the size of the remaining wound. The data points represent the % open area means ± SD. Control: n = 9 in duplicate; YC-1 30, 50 and 100 µM: n = 4 in duplicate; YC-1 treated *vs*. Control at the same time. *p < 0.05, **p < 0.01 and ***p < 0.001. (**C**) Effect of YC-1 on tube formation in HUVECs. Light microscope pictures of HUVECs seeded on Matrigel-coated wells and treated with different YC-1 concentrations for 6 h. Two representative series of images of endothelial tube structures were shown. Control HUVECs migrated to form connected tubular networks. YC-1-treated HUVECs significantly attenuated network formation. (**D**–**F**) Quantitative analysis of the total segment length, total tube length and the number of nodes were performed from photographs. Magnification: 100x. Data are expressed as means ± SD. Control cells, n = 10 in triplicate; YC-1 treated cells, n = 4 in triplicate. YC-1 treated *vs*. Control. *p < 0.05, **p < 0.01 and ***p < 0.001.

Next, the wound-healing assay was performed with early passage HUVECs that were given increasing doses of YC-1 for up to 8 hours. The migration rate of early passage HUVECs treated with YC-1 (0–100 µM) was significantly lower compared with control HUVECs (Fig. 8A,B).

Finally, early passage endothelial cells were treated with increasing concentrations of YC-1 and analyzed by angiogenesis assay (Fig. 8C–F). Images of YC-1-treated endothelial cells at 6 hours showed decreased in tube formation vs. control. Further, total segment length, total tube length, and the number of nodes fell dose-dependently. Collectively, our results demonstrate that the inhibition of HIF-1α by YC-1 impedes cell migration and tube formation in HUVECs.

To test the possibility that HIF-1α modulates miR-126 levels in endothelial cells and thus HUVECs migration and angiogenesis, we quantified miR-126-3p and miR-126-5p transcripts in HUVECs that were treated with YC-1 for 16 hours. No significant difference in miR-126-3p or miR-126-5p levels was observed (Supplemental Fig. 2). Consequently, HIF-1α-mediated changes in migration and angiogenesis are independent of miR-126 levels.

### Inhibition of microRNA-126 downregulate HIF-1α expression protein in endothelial cells

**Figure 9:**
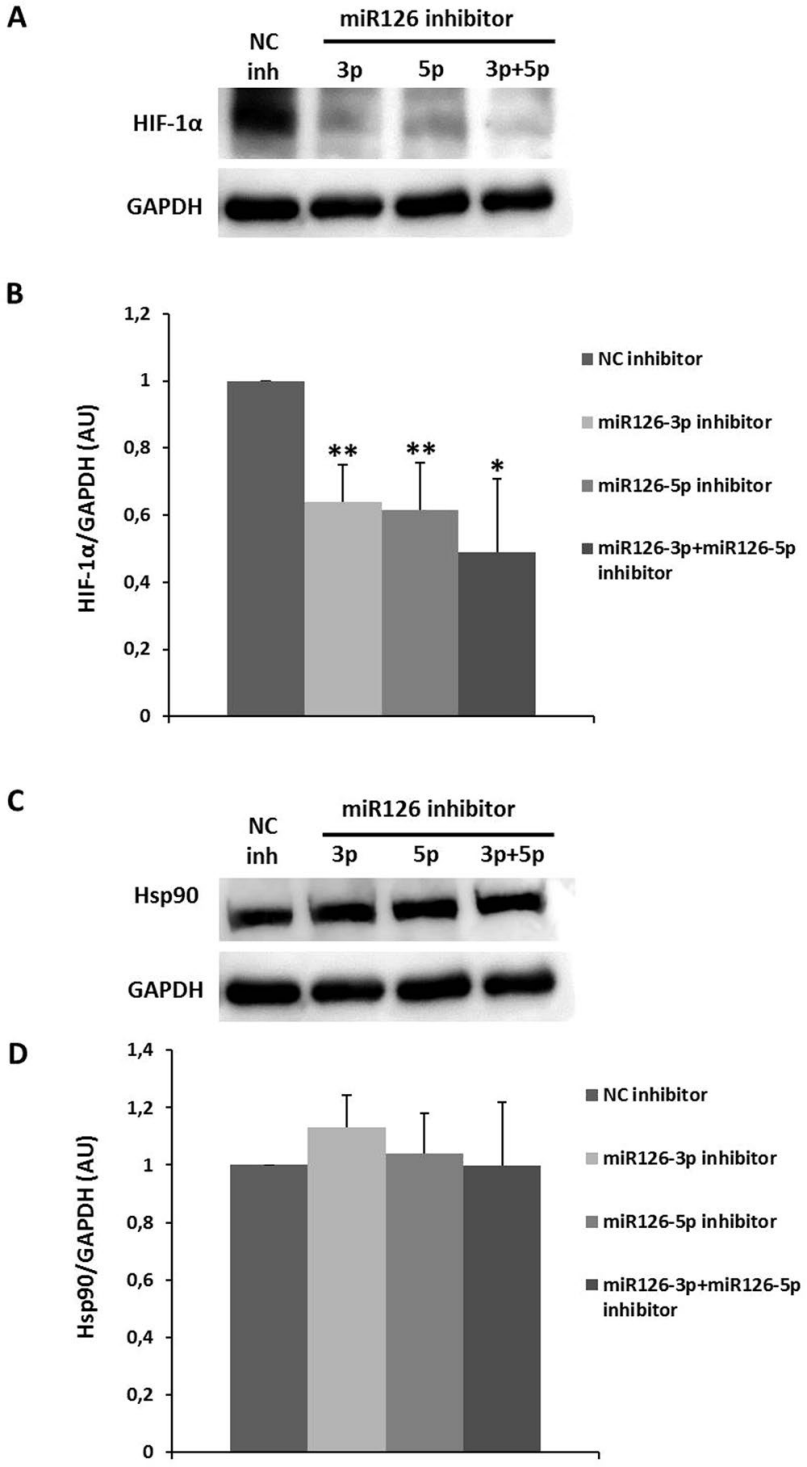
Effect of miR-126 inhibition on HIF-1α and Hsp90 protein levels in early passage HUVECs. (**A**) Representative HIF-1α western blot in early passage HUVECs transfected with negative control (NC) inhibitor, miR-126-3p strand, miR-126-5p strand or both sequence inhibitors, miR-126-3p plus miR-126-5p for 72 hours. Equal protein loading was confirmed probing with GAPDH. (**B**) The graphs present densitometric band analysis normalized to GAPDH in arbitrary units (AU). The data represent means ± SD. n = 3.Control *vs*. miR-126-transfected early passage HUVECs cells. *p < 0.05 and **p < 0.01. (**C**) Representative Hsp90 western blot in early passage HUVECs transfected with negative control (NC) inhibitor, miR-126-3p strand, miR-126-5p strand or both sequence inhibitors, miR-126-3p plus miR-126-5p for 72 hours. Equal protein loading was confirmed probing with GAPDH. (**D**) The graphs present densitometric band analysis normalized to GAPDH in arbitrary units (AU). The data represent means ± SD. n = 3.

To demonstrate the direct role of miR-126 in the HIF-1α pathway in the homeostasis maintaining in endothelial cells, early passage HUVECs were transfected with microRNA inhibitors (antimiRs), miR-126-3p, miR-126-5p or both strands, miR-126-3p plus miR-126-5p, or its corresponding anti-miR negative control (NC) inhibitors. We show that an endothelial cell-restricted miR-126-3p or miR-126-5p for 72 hours decreased HIF-1α protein compared to the corresponding anti-miR NC inhibitor (Fig. 9). Interestingly, when cells were transfected with both sequences of miR-126-3p plus miR-126-5p inhibitors during 72 hours, the HIF-1α protein was also decreased vs. the anti-miR NC inhibitor controls. Noteworthy no additive effect was seen as the reduction was similar to that of the individual miR-126 inhibitor, 3p strand and 5p strand as well as both strands together (Fig. 9A,B). No change of Hsp90 protein in early passage endothelial cells transfected with antimiRs was seen (Fig. 9C,D). These results indicate that manipulating the expression of miR-126 in early passage endothelial cells *in vitro* affected HIF-1α protein decreasing its constitutive expression. On the other hand, Hsp90 levels are maintained unalterable suggesting that Hsp90 plays no role in HIF-1α degradation mediated by miR-126.

## Discussion

Cell senescence is defined as a type of status characterized by the irreversible arrest of cell proliferation and is considered the main contributor to aging and aging-related disease^30^. In general, the senescent phenotype is distinguished by the upregulation and secretion of growth factors, extracellular matrix-degrading proteins, and proinflammatory cytokines—constituting the senescence-associated secretory phenotype (SASP)^34^—and decreased the ability to migrate^35^. At the cellular and molecular levels, telomere shortening, genomic and epigenomic damage, unbalanced mitogenic signals, and activation of tumor suppressor proteins have been related to cellular senescence^30^. Molecules that are involved in cellular senescence participate in cell cycle control and DNA repair^36^ have been used as biomarkers of senescent cells *in vitro* and *in vivo*, including the upregulation of SA-β-gal, p16, and DcR2^30^ or p21 and Cyclin D1^37^ and the loss of Lamin B1^38^. However, many common cellular markers of senescence are considered not sufficiently robust to reliably identify the number of senescent cells when present at low frequencies^39^. To avoid this distortion, we also used SA-β-gal, Cyclin D1, and Lamin B1 as senescent markers. Our data provide the first evidence that Lamin B1 and Cyclin D1—recently described^11,37,38^ as robust markers of senescence—are regulated in a replicative senescence HUVECs model.

In addition to the general changes that are associated with cell senescence described above, many cell type-specific changes have been reported. In this regard, our data provide the first evidence that the lack of HIF-1α is key in the senescence of human endothelial cells. Thereby, the senescence of vascular endothelium has been related to vascular aging^13^ and age-related vascular diseases^14^, and several mechanisms have been proposed to mediate this age-dependent endothelial dysfunction^16^. Decreased e-NOS expression^40^, redistribution of the splicing factor SRSF1^41^, endothelial progenitor cell dysfunction^42^, oxidative stress^43^, and disruption of cell junctions^11^ are all specific mechanisms of endothelial cell senescence. Besides, our results add a new mechanism in the age-mediated endothelial damage where HIF-1α plays a role in senescent endothelial cells.

There are little data on the relationship between senescence and HIF-1α. Hypoxia protects cells from oncogene-induced senescence through the downregulation of senescence markers, and HIF-1α is directly involved in the downregulation of p53 and p21 in fibroblasts^44^. Also, HIF-1α attenuates or delays premature cellular senescence, and the loss of HIF-1α induces premature senescence in fibroblasts^45^. Recent evidence also suggests that increases in HIF-1α due to hypoxic preconditioning inhibit endothelial progenitor cell senescence^46^. Consistent with these results, our findings have revealed the disappearance of HIF-1α in a replicative senescence model of human endothelial cells. Similar to previous observations that senescent cells are unable to initiate cell division or participate in tissue regeneration and wound healing^47^, our results show that senescent endothelial cells lose the capacity to undergo tube formation and participate in wound repair. Thus, the loss of standard functions that characterize senescent endothelial cells might be attributed to the disappearance of HIF-1α—we found that treatment of early passage HUVECs with an HIF-1α inhibitor significantly impaired their function, resembling a senescent phenotype. HIF-1α modulates physiological and pathological wound repair^47^ and mediates angiogenesis^48^. We speculate that the loss of HIF-1α from senescent endothelial cells is critical in endothelial homeostasis and thus in aging-associated diseases, such as endothelial dysfunction and vascular disease. Future studies should examine the mechanism resulting in lack of HIF-1α during cellular senescence.

MVs that are shed from senescent endothelial cells lack HIF-1α; however, those from early passage endothelial cells contain HIF-1α. MVs can be released from nearly all types of cells, and the proteins and miRNAs that they carry have potential diagnostic and therapeutic value^24,49^. This ability to harbor and transfer biological information renders MVs an essential route of intracellular communication and could serve as a biomarker^49^. Consequently, HIF-1α-containing MVs from early passage HUVECs could function as an essential signaling mechanism that maintains normal endothelial function and vascular homeostasis. In contrast, the absence of HIF-1α in senescent MVs suggests that this phenomenon may aggravate the dysfunction of the senescent endothelium.

Hsp90 is a chaperone involved in the activation and stabilization of many proteins avoiding proteasomal degradation. Thus, we hypothesized that Hsp90 would establish HIF-1α ubiquitination and subsequently degradation via proteasome though Hsp90/HIF-1α complex in early passage and senescent HUVECs. Several client proteins of Hsp90 have been identified, most of which are related to signal transduction, cell cycle progression, and transcriptional regulation^50^. Hsp90 is essential in the activation and stabilization of HIF-1α in hypoxia in a cell line of human microvascular endothelial^20^. In fact, Hsp90 inhibitors dissociate Hsp90 from HIF-1α and induce the degradation of HIF-1α^51^. Vascular aging increases ROS in endothelial cells and senescent HUVECs^52^, and ROS-induced Hsp90 cleavage requires iron explaining the low Hsp90 levels that we observed in senescent endothelial cells and the effects of DFO. DFO is an iron chelator and can thus halt ROS-induced Hsp90 cleavage-also, one of the effects of DFO is to maintain Hsp90 levels in senescent endothelial cells (Fig. 4). To elucidate the role of Hsp90 in HIF-1α degradation furthers experiments should be carried out. Moreover, the possibility of a direct effect of DFO on the enzymes that participate in HIF-1α metabolism should not be dismissed, because iron chelators inhibit hydroxylase activity in HIF-1α degradation^53^. DFO also sustained HIF-1α levels in senescent HUVECs, but this finding could be explained by the well-known stabilizing effect of Hsp90 on HIF-1α. The translation of Hsp90 is downregulated for specific iron chelators—opposite to our observations—but this effect has not been demonstrated for DFO^54^. It is unknown whether HIF-1α degradation depends on O_2_/VHL (von Hippel Lindau), and the disruption of Hsp90 promotes HIF-1α degradation through an O_2_/VHL-independent pathway^51^, which might be the mechanism of degradation in our senescent HUVECs (e.g., Hsp90-regulated and O_2_/VHL-independent) because our cells were not grown under hypoxic conditions. Hsp90 content becomes significantly lower in senescent HUVECs but does not change in senescent MVs, possible due to the incorporation of other client proteins of Hsp90 into MVs. Moreover, another reason why HIF-1α protein levels are abolished in senescent endothelial cells may be the higher rate of protein degradation given that DFO (a HIF-1α stabilizer) treatment in senescent HUVECs accumulates HIF-1α protein. Accordingly, HIF-1α protein half-life in senescent endothelial cells is shorter than in early passage endothelial cells. This result confirms the important role that the transcription factor HIF-1α plays in physiological and the pathophysiological conditions as an essential mediator of oxygen homeostasis^55,56^.

DFO, which maintains HIF-1α protein levels in treated cells, has no effect on the scratch assay of senescent HUVECs. This result might be attributed to the advanced state of senescence in our model versus other paradigms. The rate of PD in HUVECs in our senescent model was 97 (PD > 96; passaged 27–38 times) compared with the current knowledge on replicative senescence in endothelial cells that reported rates of PD > 44 in endothelial cells (passage 13)^54,57^, PD between 49–50 (26 passages and onwards, late senescent HUVECs)^58^, and PD approximately 45^59,60^. Thus, the senescent endothelial state was irreversible, and DFO could not have affected the migration or proliferation (endothelial function) of senescent endothelial cells. Moreover, other mediators may act in the normal function of the endothelial cells.

Our results reveal that the miR-126 levels in endothelial cells were associated with aging, where the level of miR-126 was higher in early passage endothelial cells and early passage endothelial-secreted MVs than in senescent HUVEC and senescent HUVECs-secreted MVs. Regarding this data, miR-126 is highly expressed in vascularized tissues, endothelial cells, and hematopoietic cells^61^ and is considered a prototypical endothelial-specific miRNA because miR-126 is the most highly enriched microRNA in endothelial cells^62^. Notably, the chief function of miR-126 is to regulate angiogenesis and vascular integrity and, therefore, and its expression plays a very important role in the endothelial homeostasis^26,63^. In addition, miR-126 is involved in vessel maturation and is believed to be atheroprotective^28^. Lower miR-126 levels are associated with increased inflammatory mediators^64^, and miR-126 is are downregulated in senescent human aortic endothelial cells^64^. In support of these findings, we also observed decreases in miR-126-3p and miR-126-5p levels in senescent HUVECs, which experienced a loss in function, as evidenced by the significant reduction in senescent HUVECs angiogenesis, proliferation, and migration (wound healing). The levels of the miR-126-5p strand were higher compared with the miR-126-3p strand in early passage and senescent HUVECs. Both mature miR-126 strands are critical in angiogenesis. A recent study showed that silencing of miR-126-3p, but not miR-126-5p, suppresses angiogenesis^29^. The decreases in miR-126 expression strands, miR-126-3p and miR-126-5p, in MVs from senescent HUVECs are similar, suggesting paracrine signaling between nearby cells.

Many studies in recent years have highlighted the possibility of a link between HIF-1α and miR-126. Thus, we study their possible interaction in the development of endothelial senescence—the effect of HIF-1α on microRNA-126 and also the role of miR-126 in the HIF-1α pathway in endothelial cells. In a recent work, miR-126 emerged as a new regulator in hypoxia and reperfusion^65^. Moreover, some researches show that under hypoxia conditions, VEGFA-A (a key factor in angiogenesis initiation) is regulated in endothelial cells and therefore, also miR-126, described as a direct VEGFA-A target^66^. Indeed, miR-126 plays a critical role in the regulation of CXCL12/CXCR4 pathway by inhibiting the axis in quiescent endothelial cells and HIF1α-dependent activation of this pathway in vascular injury and hypoxia^67^. In addition, it has been described changes in the expression levels of some miRNAs between them, miR-126, that stimulate Akt activation during hypoxia^68^. Additionally, the phosphatidylinositol 3 kinase (PI3K) is the activator of the Akt pathway and is targeted by miR-126^69^. Hence, the observed hypoxic deregulation of miR-126 contributes to increased VEGF expression and Akt activation^70^. Results reported that endothelial cells (HUVECs) under hyperglycemic conditions undergoing senescence and miR-126 levels are modified^71^. According to all the studies above mentioned, our work was focused in the interaction miR-126 and HIF-1α. In this regard, there were no changes in miR-126 levels in endothelial cells treated with HIF-1α inhibitor. However, the inhibition of miR-126 strands, individually (3p or 5p) or together (3p plus 5p), decrease constitutive HIF-1α protein levels, suggesting that miR-126 is upstream in the relationship between HIF-1α and miR-126 in endothelial cells. Specifically, we found that the regulation of endothelial functions is through by miR-126/HIF-1α pathway, suggesting that miR-126 modulate HIF-1α protein levels as a mechanism in the maintenance of homeostasis in the vascular environment. Beside the antimiRs effect on HIF-1α protein expression, Hsp90 levels are maintained unalterable in early passage cells transfected with miR-126 inhibitors suggesting as aforementioned that is possible the involvement of others proteins in the HIF-1α protein degradation/stability via miR-126.

**Figure 10:**
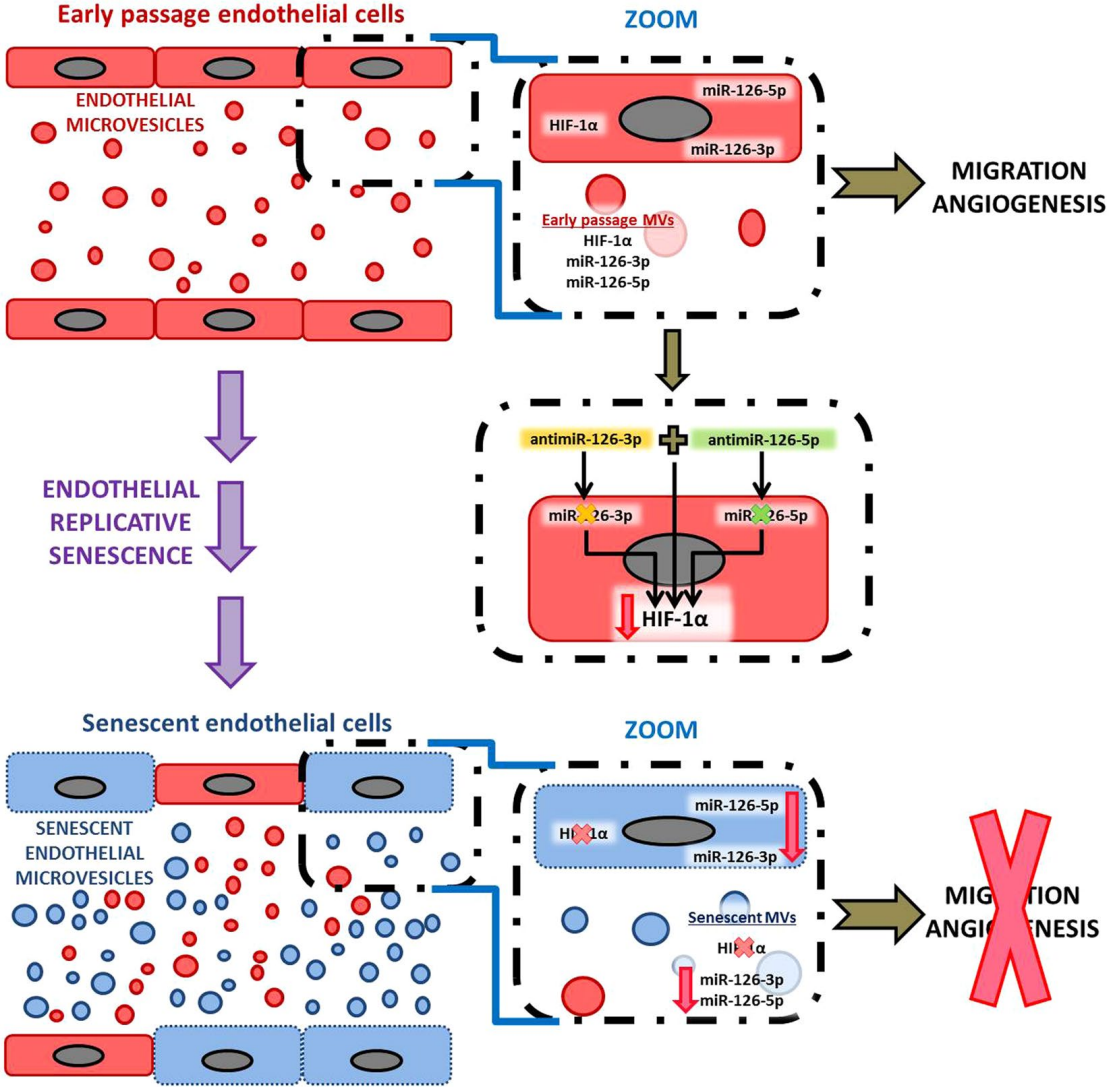
Schematic representation of miR-126 and HIF-1α signaling pathway in replicative senescence model *in vitro*.

In summary, our work demonstrates that HIF-1α is required to extend the replicative life span and implicated in the maintenance of the protective and repair functions of endothelial cells. Moreover, that miR-126 downregulated in senescent endothelial cells supports that miR-126 is an essential signaling that promotes normal endothelial function. HIF-1α and miR-126 disappear and decrease, respectively, in MVs from senescent endothelial cells, implicating them as positive regulators between cells. HIF-1α inhibition does not affect miR-126 levels, whereas the inhibition of miR-126 diminishes HIF-1α protein. Thus, the miR-126/HIF-1α pathway plays a role in the mechanisms by which endothelial function is regulated. These findings identify a new mechanism involved in replicative endothelial senescence (Fig. 10). Further *in vivo* experimental models are warranted to check the role of miR-126 in the regulation of HIF and test the potential of molecularly targeting this mechanism for therapeutic purposes.

## Methods

### HUVECs cell cultures

Human umbilical vein endothelial cells (HUVECs; CC-2517, lot number 323352 Lonza) were purchased as pooled primary cells frozen at passage 1 in a cryopreservation medium containing endothelial growth medium (EGM) with 10% heat-inactivated foetal bovine serum (FBS) (Sigma). Cultures were maintained at 37 °C in a 5% CO_2_ atmosphere at 95% humidity in EGM consisting of endothelial basal media (EBM; Lonza CC-3121) supplemented with a growth bullet kit (Lonza, CC-4133) containing Bovine Brain Extract, ascorbic acid, hydrocortisone, epidermal growth factor, gentamicin/amphotericin-B and supplemented with 10% heat-inactivated FBS. All the experiments were carried out in normoxia conditions (∼20% O_2_)^56^.

First-passage cryopreserved HUVECs were grown and serially passaged until they reached senescence, as described^72^ (the replicative senescence model) previously. The rate of population doubling (PD) occurring between passages was calculated using the formula PD = [ln {number of cells harvested} − ln {number of cells seeded})/ln2]. Cells studied within 2–8 passages (early passage; PD < 20) were regarded as early passage endothelial cells, whereas those passaged 27–38 times (PD > 96) were regarded as senescent endothelial cells.

### Cytotoxicity assay

Early passage endothelial cells seeded in 6-well plate (10^5^ cells/well) with complete endothelial growth medium (EGM supplemented with growth bullet kit and 5% heat-inactivated FBS) and treated with YC-1 (0–100 µM) during 8 hours. Later, early passage HUVECs were trypsinized, washed twice with PBS and viability was measured using 0.4% Trypan blue staining (from Countess™ Cell Counting Chamber Slides kit; ThermoFisher Scientific). Trypan blue exclusion was measured using a hemocytometer (Countess™ Automated Cell Counter; ThermoFisher Scientific) and the percentage of viable cells was determined.

### Isolation and characterization of microvesicles (MVs)

To MVs isolation, early passage and senescent HUVEC-derived (isolated from the culture medium) MVs were isolated and pooled. Briefly, cell culture supernatants were centrifuged using serial centrifugations (15 min at 3000 rpm to remove cells and cellular debris, 30 min at 14000 rpm to concentrate the MVs in Eppendorf centrifuges) based on International Society on Thrombosis and Haemostasis^73^.

MVs from HUVEC cultures were characterized following ISEV guidelines^74^, using confocal microscopy analysis for size control, flow cytometry for the quantification and analysis of membrane markers, and mass spectrometry for the detection of protein markers. Confocal microscopy showed MVs to range in size from 0.3 to 1.2 μm. An equal amount of pooled MVs from early and senescent endothelial cells were characterized in term of size using Beckman Coulter Cytomic FC 500 flow cytometer running CXP software. MVs were understood to be those events gated with a size from the side scatter (SSC) vs. forward scatter (FSC) dot-plot produced in a standardization experiment using the SPHERO™ Flow Cytometry Nano Fluorescent Size Standard Kit (Spherotech). The latter has size-calibrated fluorescent beads ranging from 0.1–1.9 μm in diameter. Events below 0.2 μm were excluded in order to distinguish true events from the background adequately; events >1.2 μm were excluded to prevent possible confusions with apoptotic bodies. Moreover, MVs from HUVEC culture medium were characterized regarding their protein profile as follow. Double-fluorescent labeling was performed to confirm the protein profile of the MVs from HUVECs. This characterization was done by incubating endothelial MVs with fluorescein isothiocyanate-conjugated (FITC) labeled monoclonal anti-CD31 (BD Pharmingen) and phycoerythrin-annexin A5 (BD Pharmingen), together in annexin A5-biding buffer (10 mM HEPES, 7.4 pH, 140 mM NaCl, 2.5 mM CaCl_2_). Isotype negative controls were also prepared. As a control for the annexin A5 labeling, a sample with fluorescein-conjugated annexin A5 using a CaCl_2_-free solution was established. To confirm the exclusion of apoptotic bodies in MVs isolations, the DNA content of the HUVEC-derived MVs was determined by incubating them with acridine orange (Invitrogen) to a final concentration of 20 mM.

Mass spectrometry analysis was performed as previously described^75^, and showed the presence of different proteins that in the ISEV guidelines are suggested as markers for the four categories of proteins that are recommended should be checked: tetraspanin and lactadherin (category 1), annexins and syntenin (category 2), cytochrome c and HSP90B1 (category 3), and fibronectin, collagen α and MMP14 (category 4). Finally, MVs were frozen and stored at −20 °C until use.

### Analysis of senescence-associated β-galactosidase (SA-β-gal) activity

SA-β-gal staining was performed using the Senescence-Galactosidase Staining Kit (MBL International Corporation, Catalog #JM-K320-250) according to the manufacturer’s protocol. In brief, endothelial cells at different passages were plated in a 12-well plate (6 × 10^4^/well) and analyzed when they reached confluency. The cells were washed with PBS and fixed in fixing solution for 12 min at room temperature. Next, they have washed once again in PBS to remove the fixing solution and incubated in freshly prepared SA-β-gal at 37 °C for 16 h without CO_2_. Afterward, SA-β- gal-positive cells (senescent cells) were identified as blue-stained cells under standard light microscopy. The number of positive cells with blue color was counted and normalized to the number of total cells in the same field. The percentage of SA-β-gal-positive cells was counted in 10 randomly selected microscopic fields (magnification 100x; 400–600 cells).

SA-β-gal activity was also measured by fluorescence microscopy. After the experiment, cells were incubated with C12FDG (fluorogenic substrate, 5-dodecanoyl-aminofluorescein di-β-D-galactopyranoside; 33 µM, Invitrogen) at 37 °C for 30 min. The images (between 7–10 for each condition) were analyzed using Image Pro-Plus software (Media Cybernetic).

### Western blot

Extracts from early passage and senescent endothelial cells, MVs from early passage and senescent HUVECs, as well as control and DFO-treated (100 µM, 8 hours, Sigma) senescent HUVECs were lysed in CytoBuster Protein Extraction Reagent lysis buffer (Millipore) containing protease and phosphatase inhibitor cocktail (Roche). The total protein content of lysates (cells and MVs) was quantified using a BCA Protein Assay Kit (Pierce), with BSA as the standard. Briefly, equal amounts of protein (10–70 μg protein/lane) were diluted with reducing sample buffer and separated by SDS/PAGE (7–10% gel) under reducing conditions. Samples were then transferred onto nitrocellulose membranes (BioRad), blocked with TBST (Tris-buffered saline, 0.1% Tween-20) containing 5% non-fat milk and then probed with the primary antibodies in the same buffer, at the following dilutions: Cyclin D1 (Thermo Scientific, Cat. No. RM-9104-SO, dilution 1/1000, 36 kDa), Lamin B1 (Abcam, ab133741, dilution 1/1000, 66 kDa), p53 (Cell Signaling Technology, #9282, dilution 1/1000, 53 kDa), p16 (Abcam, ab51243, dilution 1/1000, 16 kDa), HIF1α (BD Transduction Laboratories, Cat No. 610959, dilution 1/250, 120 kDa), Hsp90 (Cell Signaling, #4874, dilution 1/1000, 90 kDa); Anti-β-actin (Santa Cruz, sc-47778, dilution 1:2000, 43 kDa) and GAPDH (Millipore, Cat No. MAB374, dilution 1/2000, 38 kDa) were used as loading controls. After washing with TBST, the membranes were incubated with Novex horseradish peroxidase-conjugated secondary antibodies followed by 2 additional washing steps with TBST. Bands were visualized with Luminata Crescendo Western HRP substrate (Millipore). The quality of the proteins and the efficacy of protein transfer were evaluated by ATX Ponceau S red staining solution (Sigma; 09189). Finally, protein bands were quantified using Image J software (NIH) and normalized to β-actin or GAPDH in the HUVECs protein extracts and ATX Ponceau S red staining in the case of MVs. The HIF-1α protein blots were exposed for a long time (up to 30 min) to allow the detection of the absence/lack of expression of HIF-1α protein under some experimental conditions.

### Scratch assay

Culture-insert 2 well in µ-dish 35 mm was used to measure cell migration and cell proliferation rate (high ibiTreat, Ibidi). Each dish consists of two reservoirs separated by a 500 µm-thick wall. For the endothelial migration assay, an equal number of early passage and senescent endothelial cells were added into the two reservoirs of the same insert and incubated at 37 °C with 5% CO_2_. After 24 hours (when cultured cells reach 100% confluency), the insert was gently removed, creating a defined 500 µm cell-free gap. The well was filled with complete endothelial growth medium (EGM phenol red-free supplemented with growth bullet kit and 5% heat-inactivated FBS), and migration was observed using the Optika (Italy) microscope (magnification 100x). In addition, early passage endothelial cells were grown to confluency in culture-insert 2 well in µ-dish 35 mm, high ibiTreat (Ibidi) before wounding and exposed to DFO (100 µM) and different YC-1 concentrations for up to 8 hours. The wound pictures were captured by a digital camera at the same positions at 0, 1, 2, 3, 4, 5, 6, 7 and 8 hours, after which wound area was determined using ImageJ software (NIH).

### Endothelial tube formation assay

The 15 wells of a plate Ibidi μ-slide angiogenesis (Ibidi) were coated with factor-reduced Matrigel, according to the manufacturer’s instructions (Corning), and allowed to polymerize for 30 min at 37 °C. Early passage and senescent endothelial cells were seeded and grown in EGM phenol red-free supplemented with growth bullet kit and 5% heat-inactivated FBS for 6 hours in an incubator at 37 °C with 5% CO_2_. Also, early passage HUVECs (in 200 µL of EGM phenol red-free supplemented with growth bullet kit and 5% heat-inactivated FBS) were seeded in each well in the absence (negative control) or presence of various YC-1 concentrations for 6 hours in an incubator at 37 °C with 5% CO_2_ to form tubes. Network formation was observed and photographed using a phase-contrast inverted light microscope (Optika). Tube formation in the microphotographs was quantitatively analyzed by measuring the number of tubes and nodes (branching points), and tube length were measured using NIH ImageJ software with the Angiogenesis Analyzer plugin. Tubular networks were stained using Calcein AM (BD Bioscience) to visualize the cells using fluorescence microscopy.

### Real-time PCR

Total RNA was extracted from early passage and senescent HUVECs, as well as control and DFO-treated (100 µM, 8 hours) senescent HUVECs using the mirVana PARIS RNA and Native Protein Purification Kit (Ambion), according to the manufacturer’s instructions. cDNA was synthesized using the High Capacity cDNA Archive Kit (Applied Biosystems, Foster City, California, USA) with 2 µg of total RNA primed with random hexamer primers, following the manufacturer’s instructions. Real-time polymerase chain reactions (PCR) were performed on ABI Prism 7500 sequence detection PCR system (TaqMan® Universal Master Mix II, No AmpErase® UNG; Applied Biosystems) according to the manufacturer’s protocol. Assay ID used was: HIF1-α, Hs00153153_m1. Data were normalized with HPRT1 (assay ID: Hs02800695_m1). The mRNA copy numbers were calculated for each sample by the instrument software using comparative threshold (Ct) value. Relative fold-change was determined using the 2−ΔΔCt method, with early passage HUVECs as the baseline and normalized to HPRT1 expression.

### Real-time PCR of mature microRNAs

Total RNA was extracted from HUVECs, from MVs of early passage and senescent HUVECs, and from control and YC-1-treated early passage HUVECs. Total RNA was isolated using the mirVana PARIS RNA and Native Protein Purification Kit (Ambion) according to the manufacturer’s instructions. miRNA targets were reverse transcribed with a pool of the RT primers taken from TaqMan Small RNA Assays (Applied Biosystems) and total RNA using the TaqMan MicroRNA Reverse Transcription Kit (Applied Biosystems). A reaction master mix was assembled, spiked with reaction synthetic miR-39-3p (Ambion, ID: MC10956) and added to each RNA sample. Reaction tubes were kept on ice for at least 5 min, followed by incubation in a thermal cycler at 16 °C for 30 min, 42 °C for 30 min, 85 °C for 5 min, and kept at 4 °C. A micro-NTC (no-template control) that contained no sample RNA was included among these reverse-transcription reactions.

Subsequently, quantitative real-time PCR was performed in a PCR reaction containing 20x TaqMan miR Assay in which PCR primers and probes (5′-FAM) were contained, 2x TaqMan Universal Master mix no UNG (Applied Biosystems) and RT product. The reaction mix was first incubated at 95 °C for 10 min followed by 40 cycles of 95 °C for 15 s and 60 °C for 1 min. Assay ID used were: microRNA-126-3p, 002228: microRNA-126-5p, 000451; U6 snRNA, 001973 and microRNA-39-3p, 000200, and were purchased by Applied Biosystems. The relative amount of each miRNA was calculated using the comparative threshold (Ct) method with ΔCt, Ct(miRNA)–Ct(U6 snRNA) in endothelial cells and ΔCt, Ct(miRNA)–Ct(microRNA39-3p) in MVs. The miRs copy numbers were calculated for each sample by the instrument software using Ct value. Relative quantification of miRNA expression was calculated with the 2−ΔΔCt method, with early passage HUVECs as the baseline and normalized to U6 snRNA (internal control miRs) expression, and with early passage MVs as the baseline and normalized to microRNA39-3p exogenous sequence (spike-in) for MVs.

### Cell transfection and miR-126 silencing

MiR-126 silencing in cultured cells was performed using either a predesigned mirVana miRNA inhibitor corresponding to the hsa-miR126-3p and hsa-miR-126-5p sequence (Assays ID: MH12841 and MH10401, respectively; Ambion) or an anti-miR negative control#1 (AM17012; Ambion). Transfections were performed on cells that were 80% confluent for 6 h with Lipofectamine RNAiMAX reagent (Invitrogen) according to the manufacturer’s guidelines. Then, HUVECs were lysed after 72 hours of being transfected, and the lysate extraction was analyzed by Western blot.

### Statistical analysis

Data are represented as the mean ± SD. Two groups comparison was calculated by the Student’s t-test (2-tailed p-values) or Mann-Whitney tests for the comparison of normally or not normally distributed variables, respectively. Otherwise, statistical significance was determined with ANOVA followed by the Kruskal-Wallis test. Values less than 0.05 were deemed to correspond to differences between means of two independent experiments that are statistically significant. P-value < 0.05 was considered as statistically significant wherein *p < 0.05, **p < 0.01 and ***p < 0.001. GraphPad Prism 6 was used to determine statistical significance.

## Supplementary information

Supplemental figure legends

## References

[1] Rajendran P (2013). The vascular endothelium and human diseases. Int J Biol Sci.

[2] Carrecedo J, R.-C. R., Alique M. & Ramírez-Chamond R. *Endothelial cell senescence in the pathogenesis of endothelial dysfunction*. 1–16 (Intech, 2018).

[3] Luna C (2016). Aging-associated oxidized albumin promotes cellular senescence and endothelial damage. *Clinical Interventions in*. Aging.

[4] Longatto Filho Adhemar, Lopes José Manuel, Schmitt Fernando C. (2010). Angiogenesis and Breast Cancer. Journal of Oncology.

[5] Guo L (2018). CD163+ macrophages promote angiogenesis and vascular permeability accompanied by inflammation in atherosclerosis. J Clin Invest.

[6] Lamalice L, Le Boeuf F, Huot J (2007). Endothelial cell migration during angiogenesis. Circ Res.

[7] Carmeliet P (2005). Angiogenesis in life, disease and medicine. Nature.

[8] Oklu Rahmi, Walker Thomas G., Wicky Stephan, Hesketh Robin (2010). Angiogenesis and Current Antiangiogenic Strategies for the Treatment of Cancer. Journal of Vascular and Interventional Radiology.

[9] Carmeliet P (2003). Angiogenesis in health and disease. Nat Med.

[10] El Assar M, Angulo J, Rodríguez-Mañas L (2013). Oxidative stress and vascular inflammation in aging. Free Radic Biol Med.

[11] Krouwer VJ, Hekking LH, Langelaar-Makkinje M, Regan-Klapisz E, Post JA (2012). Endothelial cell senescence is associated with disrupted cell-cell junctions and increased monolayer permeability. Vasc Cell.

[12] Donato AJ, Morgan RG, Walker AE, Lesniewski LA (2015). Cellular and molecular biology of aging endothelial cells. J Mol Cell Cardiol.

[13] Minamino T (2004). Vascular cell senescence and vascular aging. J Mol Cell Cardiol.

[14] Yin H, Pickering JG (2016). Cellular Senescence and Vascular Disease: Novel Routes to Better Understanding and Therapy. Can J Cardiol.

[15] Alique M (2017). Microvesicles from the plasma of elderly subjects and from senescent endothelial cells promote vascular calcification. Aging (Albany NY).

[16] Erusalimsky JD, Skene C (2009). Mechanisms of endothelial senescence. Exp Physiol.

[17] de Magalhães JP (2004). From cells to ageing: a review of models and mechanisms of cellular senescence and their impact on human ageing. Exp Cell Res.

[18] Semenza GL (1999). Regulation of mammalian O_2_ homeostasis by hypoxia-inducible factor 1. Annu Rev Cell Dev Biol.

[19] Moroz E (2009). Real-time imaging of HIF-1alpha stabilization and degradation. PLoS One.

[20] Minet E (1999). Hypoxia-induced activation of HIF-1: role of HIF-1alpha-Hsp90 interaction. FEBS Lett.

[21] Masoud GN, Li W (2015). HIF-1α pathway: role, regulation and intervention for cancer therapy. Acta Pharm Sin B.

[22] Freyssinet JM (2003). Cellular microparticles: what are they bad or good for?. J Thromb Haemost.

[23] França CN, Izar MC, Amaral JB, Tegani DM, Fonseca FA (2015). Microparticles as potential biomarkers of cardiovascular disease. Arq Bras Cardiol.

[24] Yáñez-Mó M (2015). Biological properties of extracellular vesicles and their physiological functions. J Extracell Vesicles.

[25] Vince RV, Chrismas B, Midgley AW, McNaughton LR, Madden LA (2009). Hypoxia mediated release of endothelial microparticles and increased association of S100A12 with circulating neutrophils. Oxid Med Cell Longev.

[26] Wang S (2008). The endothelial-specific microRNA miR-126 governs vascular integrity and angiogenesis. Dev Cell.

[27] Bartel DP (2004). MicroRNAs: genomics, biogenesis, mechanism, and function. Cell.

[28] Schober A (2014). MicroRNA-126-5p promotes endothelial proliferation and limits atherosclerosis by suppressing Dlk1. Nat Med.

[29] Zhou J (2013). Regulation of vascular smooth muscle cell turnover by endothelial cell-secreted microRNA-126: role of shear stress. Circ Res.

[30] Campisi J (2013). Aging, cellular senescence, and cancer. Annu Rev Physiol.

[31] Campisi J (2005). Senescent cells, tumor suppression, and organismal aging: good citizens, bad neighbors. Cell.

[32] Raposo G, Stoorvogel W (2013). Extracellular vesicles: exosomes, microvesicles, and friends. J Cell Biol.

[33] Hsu HK (2003). YC-1 inhibits proliferation of human vascular endothelial cells through a cyclic GMP-independent pathway. Biochem Pharmacol.

[34] Coppé JP, Desprez PY, Krtolica A, Campisi J (2010). The senescence-associated secretory phenotype: the dark side of tumor suppression. Annu Rev Pathol.

[35] Ruiz-Torres A, Lozano R, Melón J, Carraro R (2003). Age-dependent decline of *in vitro* migration (basal and stimulated by IGF-1 or insulin) of human vascular smooth muscle cells. J Gerontol A Biol Sci Med Sci.

[36] van Deursen JM (2014). The role of senescent cells in ageing. Nature.

[37] Burton DG (2007). Cyclin D1 overexpression permits the reproducible detection of senescent human vascular smooth muscle cells. Ann N Y Acad Sci.

[38] Freund A, Laberge RM, Demaria M, Campisi J (2012). Lamin B1 loss is a senescence-associated biomarker. Mol Biol Cell.

[39] Lawless C (2010). Quantitative assessment of markers for cell senescence. Exp Gerontol.

[40] Matsushita H (2001). eNOS activity is reduced in senescent human endothelial cells: Preservation by hTERT immortalization. Circ Res.

[41] Blanco FJ, Bernabéu C (2012). The Splicing Factor SRSF1 as a Marker for Endothelial Senescence. Front Physiol.

[42] Williamson K, Stringer SE, Alexander MY (2012). Endothelial progenitor cells enter the aging arena. Front Physiol.

[43] Unterluggauer H, Hampel B, Zwerschke W, Jansen-Dürr P (2003). Senescence-associated cell death of human endothelial cells: the role of oxidative stress. Exp Gerontol.

[44] Kilic Eren M, Tabor V (2014). The role of hypoxia inducible factor-1 alpha in bypassing oncogene-induced senescence. PLoS One.

[45] Welford SM (2006). HIF1alpha delays premature senescence through the activation of MIF. Genes Dev.

[46] Lee SH (2013). Hypoxia inhibits cellular senescence to restore the therapeutic potential of old human endothelial progenitor cells via the hypoxia-inducible factor-1α-TWIST-p21 axis. Arterioscler Thromb Vasc Biol.

[47] Ruthenborg RJ, Ban JJ, Wazir A, Takeda N, Kim JW (2014). Regulation of wound healing and fibrosis by hypoxia and hypoxia-inducible factor-1. Mol Cells.

[48] Giannarelli C (2014). Alternatively Spliced Tissue Factor Promotes Plaque Angiogenesis Through the Activation of Hypoxia-Inducible Factor-1 alpha and Vascular Endothelial Growth Factor Signaling. Circulation.

[49] Revenfeld AL (2014). Diagnostic and prognostic potential of extracellular vesicles in peripheral blood. Clin Ther.

[50] Li J, Soroka J, Buchner J (2012). The Hsp90 chaperone machinery: conformational dynamics and regulation by co-chaperones. Biochim Biophys Acta.

[51] Liu YV, Semenza GL (2007). RACK1 vs. HSP90: competition for HIF-1 alpha degradation vs. stabilization. Cell Cycle.

[52] Beck R (2012). Hsp90 is cleaved by reactive oxygen species at a highly conserved N-terminal amino acid motif. PLoS One.

[53] Semenza GL (2007). Hypoxia-inducible factor 1 (HIF-1) pathway. Sci STKE.

[54] Sidarovich V (2015). Translational downregulation of HSP90 expression by iron chelators in neuroblastoma cells. Mol Pharmacol.

[55] Semenza GL (2000). HIF-1: mediator of physiological and pathophysiological responses to hypoxia. J Appl Physiol (1985).

[56] Kumar H, Choi DK (2015). Hypoxia Inducible Factor Pathway and Physiological Adaptation: A Cell Survival Pathway?. Mediators Inflamm.

[57] Kim KS (2007). Regulation of replicative senescence by insulin-like growth factor-binding protein 3 in human umbilical vein endothelial cells. Aging Cell.

[58] AbuBakar S, Shu MH, Johari J, Wong PF (2014). Senescence affects endothelial cells susceptibility to dengue virus infection. Int J Med Sci.

[59] Mariotti M, Castiglioni S, Bernardini D, Maier JA (2006). Interleukin 1 alpha is a marker of endothelial cellular senescent. Immun Ageing.

[60] Korybalska K (2013). Recovery of senescent endothelial cells from injury. J Gerontol A Biol Sci Med Sci.

[61] Chamorro-Jorganes A, Araldi E, Suárez Y (2013). MicroRNAs as pharmacological targets in endothelial cell function and dysfunction. Pharmacol Res.

[62] Yamakuchi Munekazu, Hashiguchi Teruto (2018). Endothelial Cell Aging: How miRNAs Contribute?. Journal of Clinical Medicine.

[63] van Solingen C (2009). Antagomir-mediated silencing of endothelial cell specific microRNA-126 impairs ischemia-induced angiogenesis. J Cell Mol Med.

[64] Rippe C (2012). MicroRNA changes in human arterial endothelial cells with senescence: relation to apoptosis, eNOS and inflammation. Exp Gerontol.

[65] Guenther SP, Schrepfer S (2016). miR-126: a potential new key player in hypoxia and reperfusion?. Ann Transl Med.

[66] Chistiakov DA, Orekhov AN, Bobryshev YV (2016). The role of miR-126 in embryonic angiogenesis, adult vascular homeostasis, and vascular repair and its alterations in atherosclerotic disease. J Mol Cell Cardiol.

[67] Bijkerk R (2014). Hematopoietic microRNA-126 protects against renal ischemia/reperfusion injury by promoting vascular integrity. J Am Soc Nephrol.

[68] Kalinowski L (2016). Posttranscriptional and transcriptional regulation of endothelial nitric-oxide synthase during hypoxia: the role of microRNAs. Cell Mol Biol Lett.

[69] Guo C (2008). The noncoding RNA, miR-126, suppresses the growth of neoplastic cells by targeting phosphatidylinositol 3-kinase signaling and is frequently lost in colon cancers. Genes Chromosomes Cancer.

[70] Ye P, Liu J, He F, Xu W, Yao K (2014). Hypoxia-induced deregulation of miR-126 and its regulative effect on VEGF and MMP-9 expression. Int J Med Sci.

[71] Olivieri F (2014). Age- and glycemia-related miR-126-3p levels in plasma and endothelial cells. Aging (Albany NY).

[72] Kurz DJ, Decary S, Hong Y, Erusalimsky JD (2000). Senescence-associated (beta)-galactosidase reflects an increase in lysosomal mass during replicative ageing of human endothelial cells. J Cell Sci.

[73] Robert S (2009). Standardization of platelet-derived microparticle counting using calibrated beads and a Cytomics FC500 routine flow cytometer: a first step towards multicenter studies?. J Thromb Haemost.

[74] Lötvall J (2014). Minimal experimental requirements for definition of extracellular vesicles and their functions: a position statement from the International Society for Extracellular Vesicles. J Extracell Vesicles.

[75] Bodega G (2017). The Antioxidant Machinery of Young and Senescent Human Umbilical Vein Endothelial Cells and Their Microvesicles. Oxid Med Cell Longev.

