# Supplementary material for: MicroRNA-126 regulates Hypoxia-Inducible Factor-1α which inhibited migration, proliferation, and angiogenesis in replicative endothelial senescence
Source: Sci Rep. 2019 May 14;9:7381. doi: 10.1038/s41598-019-43689-3 (PMC6517399; doi:10.1038/s41598-019-43689-3)

MicroRNA-126 regulates Hypoxia-Inducible Factor-1α which inhibited migration, proliferation, and angiogenesis in replicative endothelial senescence

Matilde Alique, PhD[1*], Guillermo Bodega, PhD [2], Chiara Giannarelli, MD, PhD[3,4,5], Julia Carracedo, MD, PhD[6,7&] and Rafael Ramírez, MD, PhD [1&]

[1]Departamento Biología de Sistemas, Facultad de Medicina y Ciencias de la Salud, Universidad de Alcalá, Alcalá de Henares, Madrid, Spain.

[2]Departamento de Biomedicina y Biotecnología, Facultad de Biología, Química y Ciencias Ambientales, Universidad de Alcalá. Alcalá de Henares, Madrid, Spain.

[3]Cardiovascular Research Center, [4]Institute for Genomics and Multiscale Biology, [5] Precision Immunology Institute, Icahn School of Medicine at Mount Sinai, One Gustave L. Levy Place, New York, NY, USA.

[6]Departamento de Genética, Fisiología y Microbiología, Facultad de Biología, Universidad Complutense de Madrid, [7]Instituto de Investigación Sanitaria Hospital 12 de Octubre (imas12), Madrid, Spain.

& These authors share senior authorship.

*** Corresponding author:** Dr. Matilde Alique. Universidad de Alcalá. Facultad de Medicina y Ciencias de la Salud. Departamento Biología de Sistemas. E-28871 Alcalá de Henares, Madrid, Spain. E-mail: matilde.alique@uah.es

SUPPLEMENTAL FIGURES:

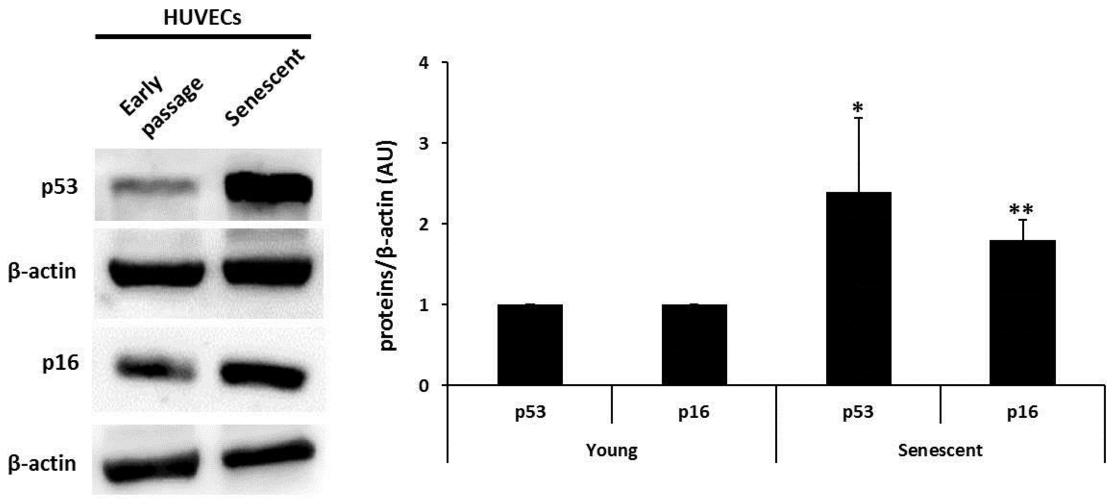

Figure 1S

Supplemental figure 1: Traditional cellular markers of senescence in HUVEC. HUVECs develop a senescence phenotype with increasing passage number in vitro. p53 and p16 representative Western blots in early passage and senescent HUVEC pools. Equal protein loading was confirmed probing with β-actin. The graph presents densitometric band analysis normalized to β-actin in arbitrary units (AU). The data represent means±SD and are expressed as fold induction with respect to control values (early passage cells). Early passage endothelial cells n=3 pools; senescent endothelial cells n=3 pools. $^*p<0.05$ and $^{**}p<0.01$. Early passage vs. senescent HUVEC cells. In the figure graphs, the early passage is called young.

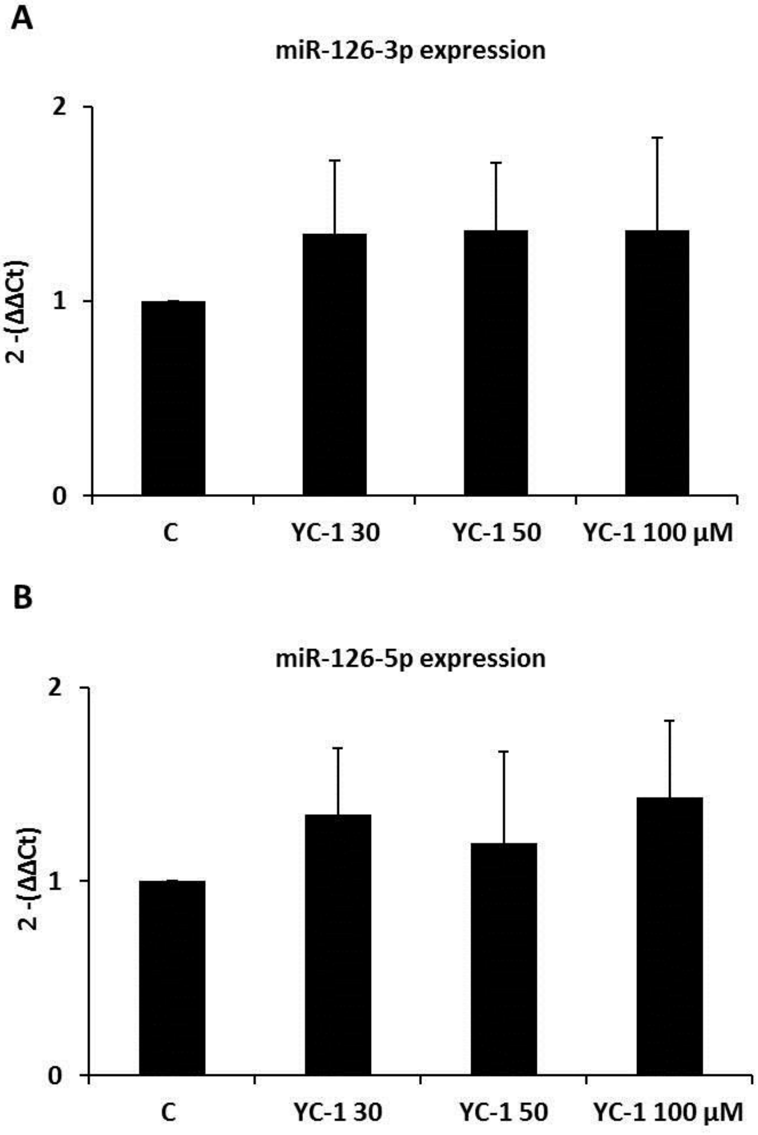

Figure 2S

Supplemental figure 2: miR-126-3p and miR-126-5p in YC-1-treated early passage **HUVEC.** qPCR analysis of miR-126-3p (A) and miR-126-5p (B) was performed in YC-1-treated early passage HUVEC using the ΔCt method; U6 snRNA was used for normalization. HUVEC were YC-1-treated with different doses (30, 50, 100 μM) for 16h. The data represent

means±SD and are expressed as fold induction with respect to control values (control HUVEC), n=4.

Uncropped blots used in Figure 1

Cyclin D1

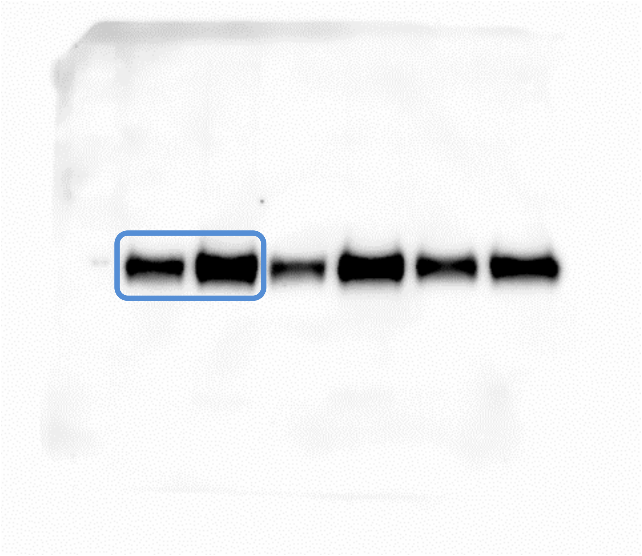

β-actin (Cyclin D1)

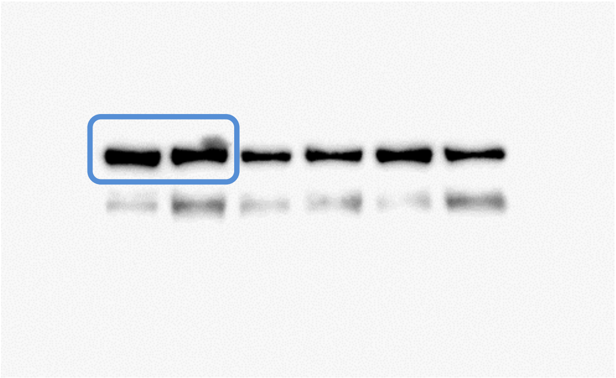

Lamin B1

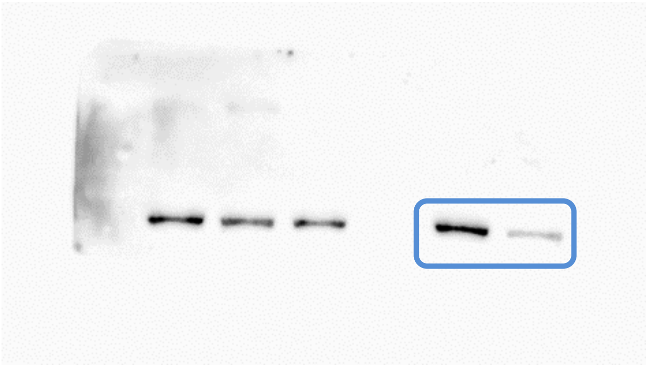

β-actin (Lamin B1)

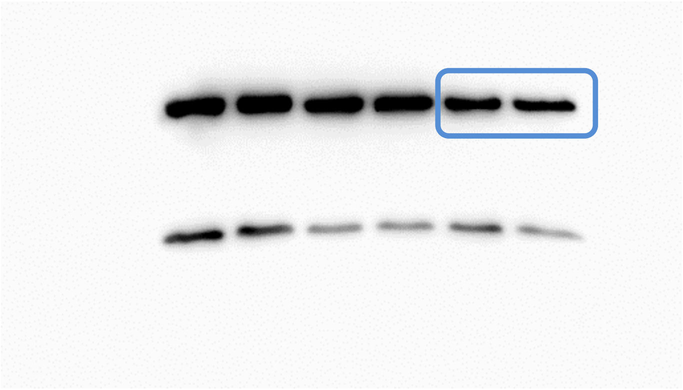

Uncropped blots used in Figure 1S

p53

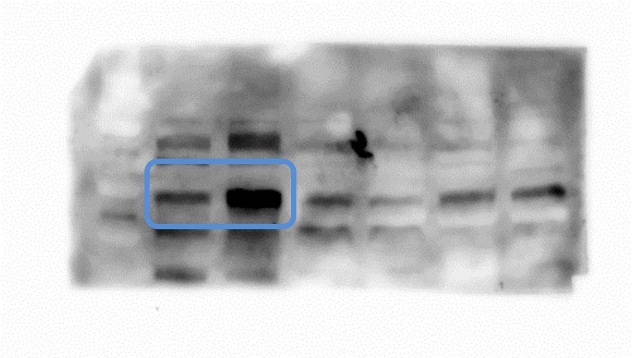

p16

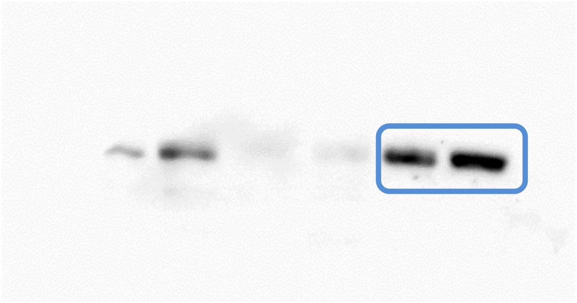

β-actin (p53 and p16)

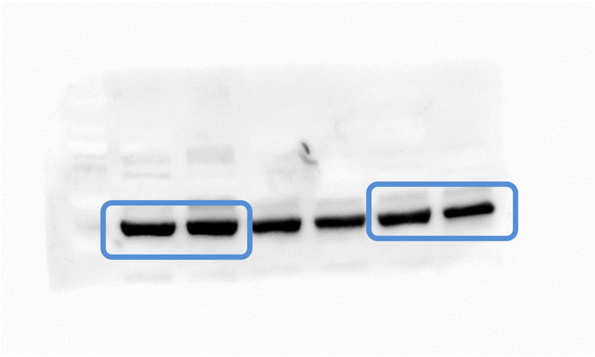

Uncropped blots used in Figure 7

HIF-1α

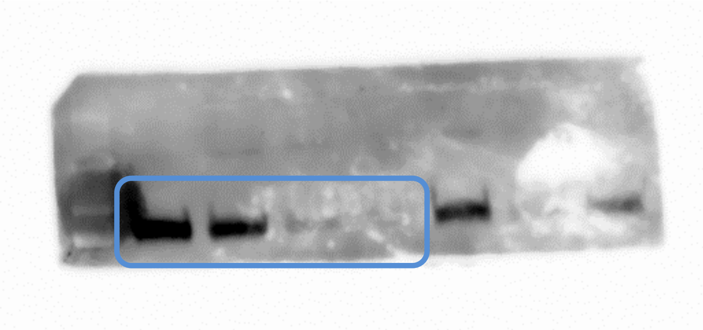

GAPDH (HIF-1α and Hsp90)

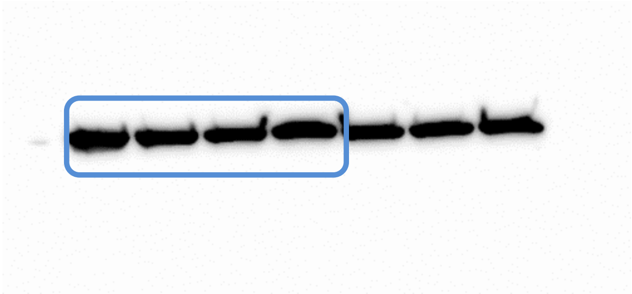

Hsp90

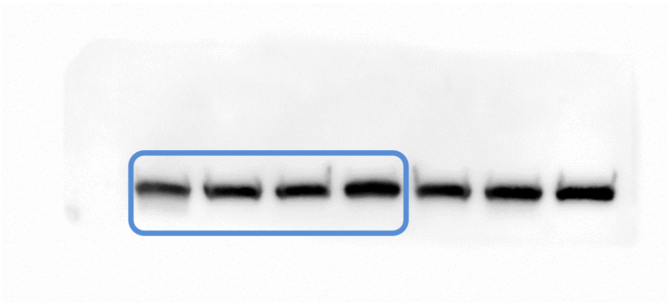

GAPDH (HIF-1α and Hsp90)

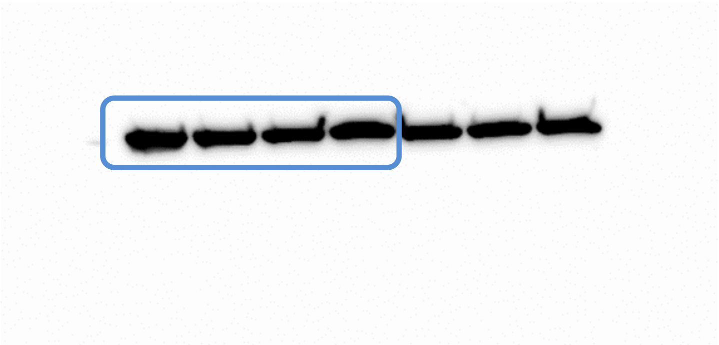

Uncropped blots used in Figure 3

HIF-1α

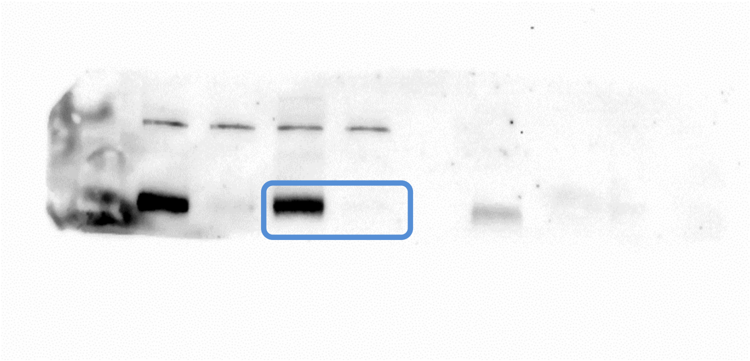

GAPDH

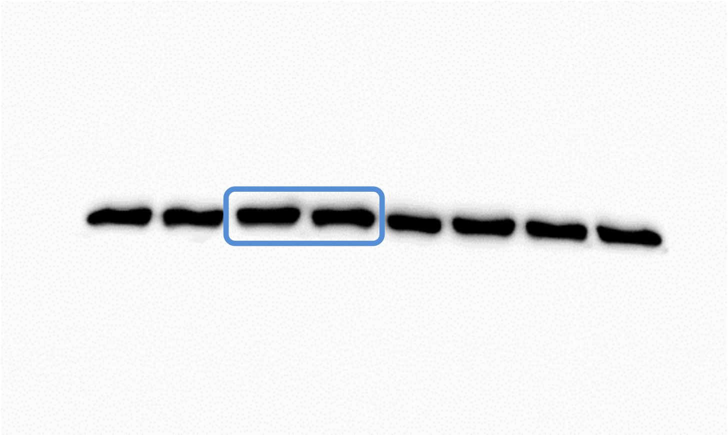

Hsp90

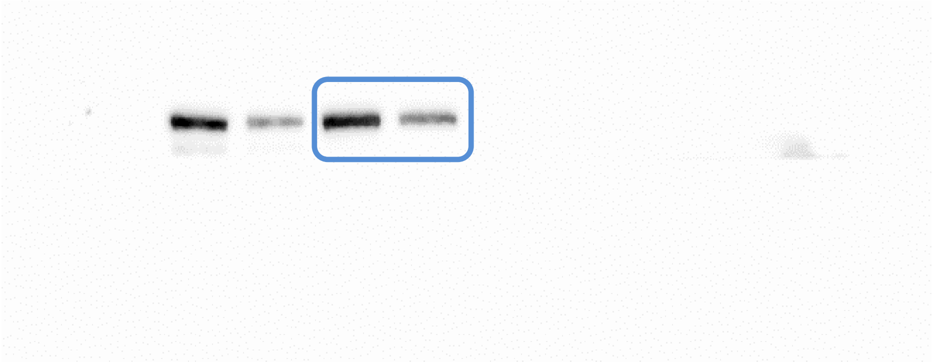

GAPDH

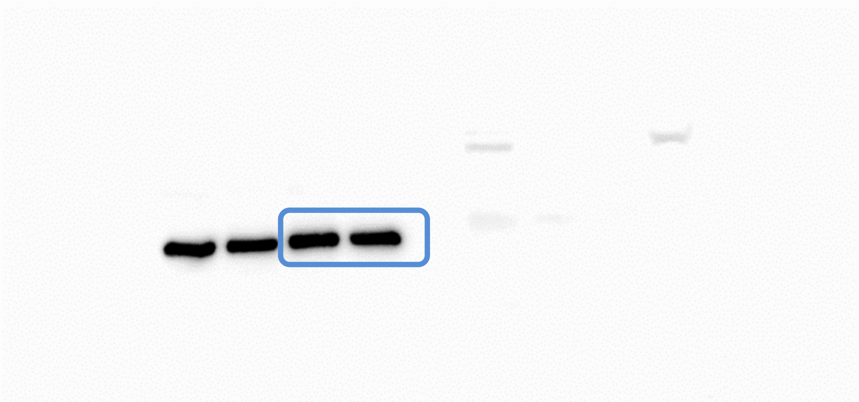

HIF-1α

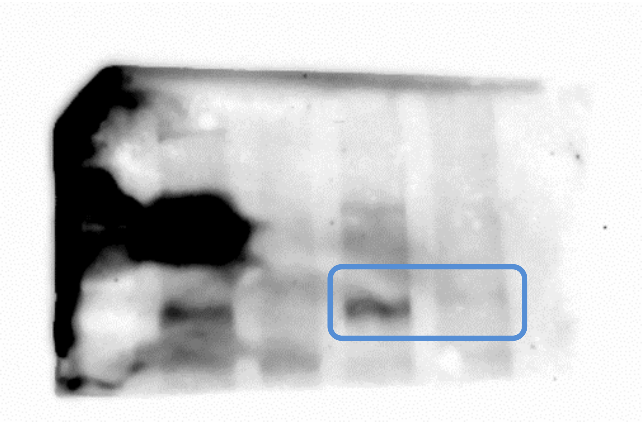

Ponceau red staining

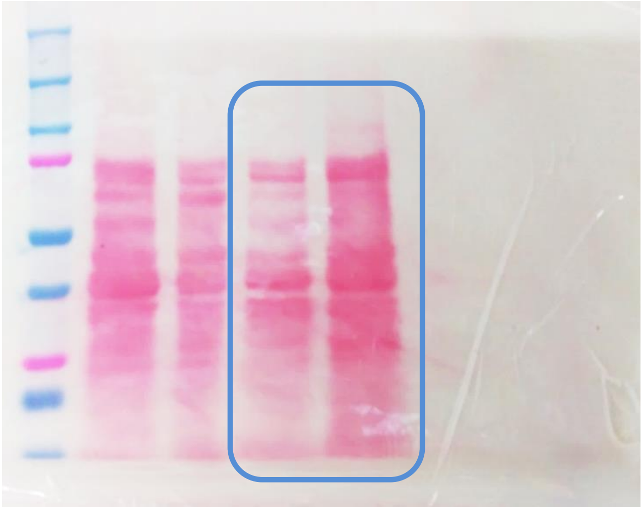

Hsp90

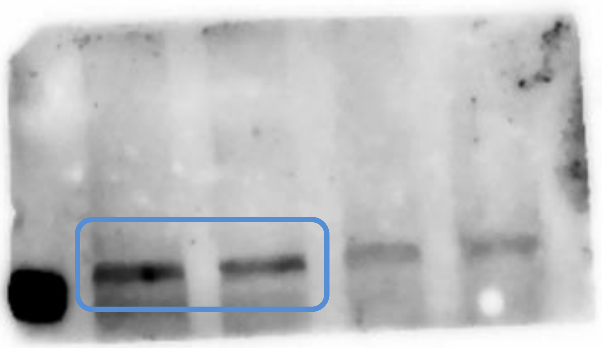

Ponceau red staining

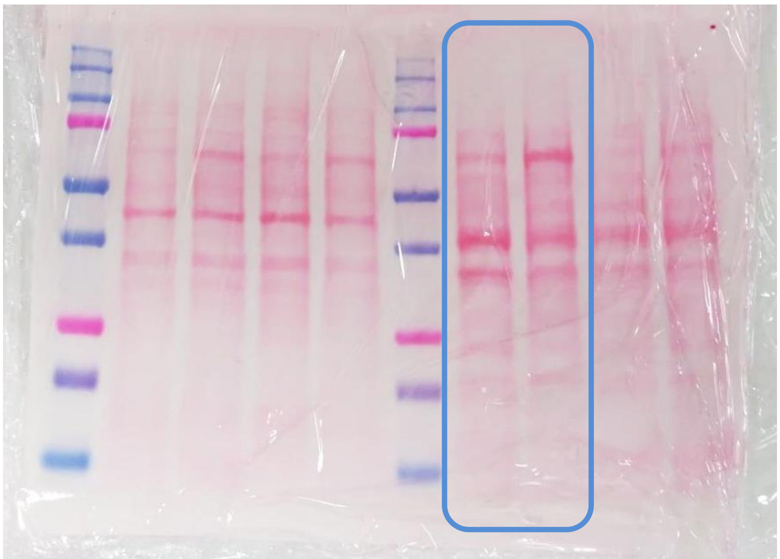

Uncropped blots used in Figure 4

HIF-1α

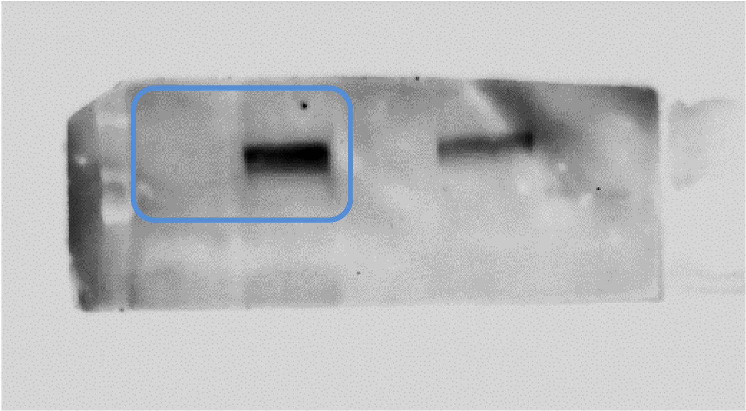

GAPDH

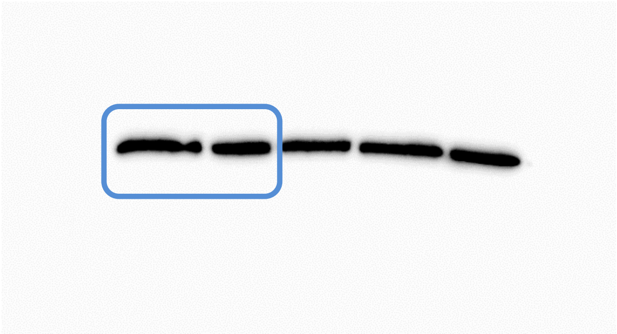

Hsp90

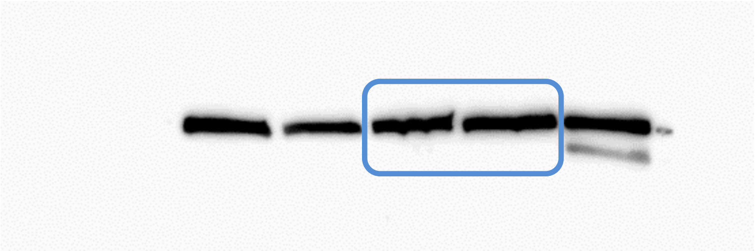

GAPDH

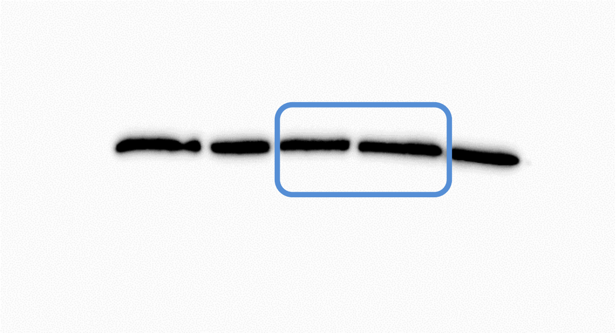

Uncropped blots used in Figure 9

Second lane: non-transfected HUVEC cells

HIF-1α

Long exposure

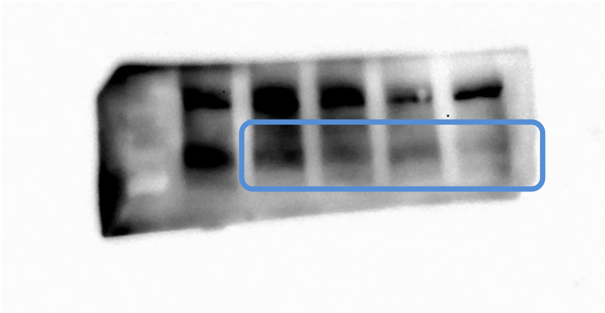

Short exposure

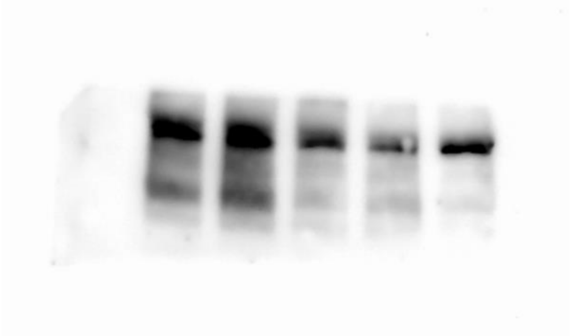

GAPDH

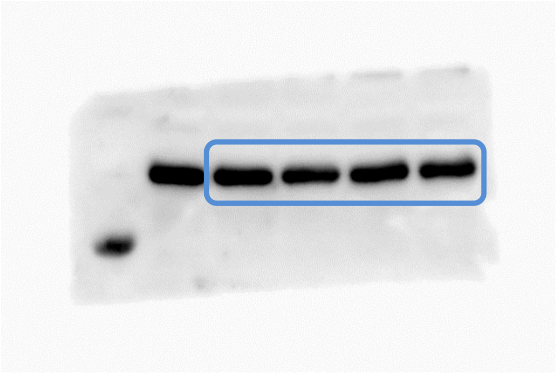

Hsp90

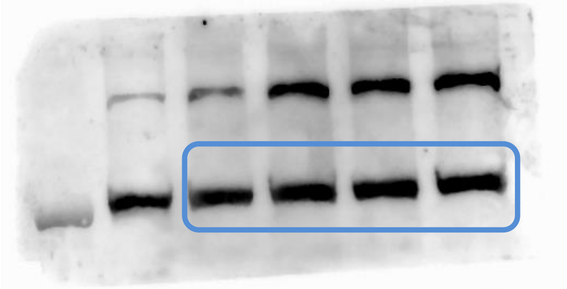

GAPDH

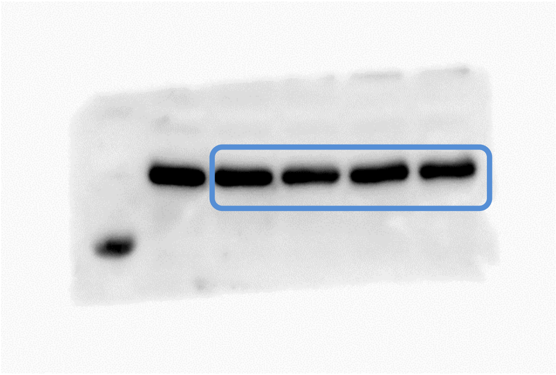

Supplement: Supplementary file 1 — Supplemental figure legends [file 41598_2019_43689_MOESM1_ESM.pdf]
